# Using Transcriptome Analysis to Explore Gray Mold Resistance-Related Genes in Onion (*Allium* *cepa* L.)

**DOI:** 10.3390/genes13030542

**Published:** 2022-03-18

**Authors:** Hyun-Min Lee, Jee-Soo Park, So-Jeong Kim, Seung-Gyu Kim, Young-Doo Park

**Affiliations:** Department of Horticultural Biotechnology, Kyung Hee University, 1732 Deogyoung-daero, Giheung-gu, Yongin-si 17104, Gyeonggi-do, Korea; ahawhgek@naver.com (H.-M.L.); jeesoo_92@naver.com (J.-S.P.); coalla110@naver.com (S.-J.K.); sg940803@gmail.com (S.-G.K.)

**Keywords:** *Allium cepa*, gene expression, gray mold resistant, in vitro inoculation, transcriptome analysis

## Abstract

Gray mold disease caused by *Botrytis* in onions (*Allium cepa* L.) during growth and storage negatively affects their yield and quality. Exploring the genes related to gray mold resistance in onion and their application to the breeding of resistant onion lines will support effective and ecological control methods of the disease. Here, the genetic relationship of 54 onion lines based on random amplified polymorphic DNA (RAPD) and in vitro-cultured onion lines infected with gray mold were used for screening resistance and susceptibility traits. Two genetically related onion lines were selected, one with a resistant and one with a susceptible phenotype. In vitro gray mold infection was repeated with these two lines, and leaf samples were collected for gene expression studies in time series. Transcript sequences obtained by RNA sequencing were subjected to DEG analysis, variant analysis, and KEGG mapping. Among the KEGG pathways, ‘α-linoleic acid metabolism’ was selected because the comparison of the time series expression pattern of *Jasmonate resistant 1* (*JAR1*), *Coronatine-insensitive protein 1* (*COI 1*), and transcription factor *MYC2* (*MYC2*) genes between the resistant and susceptible lines revealed its significant relationship with gray-mold-resistant phenotypes. Expression pattern and SNP of the selected genes were verified by quantitative real-time PCR and high-resolution melting (HRM) analysis, respectively. The results of this study will be useful for the development of molecular marker and finally breeding of gray-mold-resistant onions.

## 1. Introduction

Onion is an important vegetable that ranks second among the world’s most produced crops [1]. In 2019, the global onion production was 99 million tons [2]. Onions are used as a food ingredient and have a positive effect on human health. Their high phenol content, flavonoids, and organo-sulfur compounds act as antioxidants to scavenge free radicals [3,4,5,6] and protect the body against several diseases, including cancer [7,8,9,10]. Onions have a biennial life cycle, high inbreeding depression, and cross-pollinated nature, hindering the breeding for traits such as disease resistance [11].

A variety of fungi in the genus *Botrytis* cause diseases during onion cultivation and storage, leading to economic losses. For example, *Botrytis aclada* Fresen causes sclerotial neck rot in onions [12], and *Botrytis byssoidea* J.C. Walker causes mycelial neck rot [12]. *Botrytis squamosa* J.C. Walker causes small sclerotial neck rot [12] and onion leaf blight [13] in white onion cultivars. *Botrytis cinerea* Pers. causes leaf spot disease and discoloration in onion bulbs [14] and is sometimes found in rotten neck tissues of onions [15].

Fungicides [16,17] and cultural control methods [18] are used to control *Botrytis* species during onion production. Biological controls also include fungi [19] and bacteria [20] that show antagonistic activity against *Botrytis* species. These methods are short-term and laborious and increase the resistance of the pathogens. There is a need to breed onions that are resistant to *Botrytis* species, and research conducted to screen and breed *Botrytis*-resistant onion lines has been reported [21,22,23].

The expression of genes related to specific environments is essential for breeding crops with specific traits such as disease resistance. High-throughput sequencing of the genome, using as next-generation sequencing (NGS) and RNA sequencing for transcriptome characterization, paved the way for marker development [24]. Owing to the large genome size (16.3 Gb) [25], several studies have been conducted to construct a reference through RNA sequencing of onions [26,27]. In addition, studies on genes related to various traits in onions have also been conducted using RNA sequencing. RNA sequencing studies on genes related to various bulb colors in onions, such as a white bulb color [28], chartreuse bulb color [29], and dark-red bulb color [30], have been reported. In addition, studies on genes involved in cytoplasmic male sterility [31] and genes involved in fertility restoration of cytoplasmic male sterility [32] have been conducted. Genes related to tolerance to abiotic stress caused by external environmental conditions, such as cold stress [33] and drought stress [34] were also obtained through RNA sequencing.

To combat pathogen infection, plants utilize both pre-existing and induced defense strategies [35]. The production of jasmonic acid and its derivatives, collectively known as jasmonates (JAs), is an important defense response [36]. Signaling molecules, such as JAs, induce the expression of a series of genes under environmental stress, including pathogen infections [37,38]. JAs are known to be involved in resistance to infections by necrotrophic pathogens, especially *Botrytis* species [39,40].

This study aimed to characterize and analyze the expression of genes that are related to the resistance to *Botrytis* species in onions. Resistant and susceptible onion lines were screened by infecting onions with *Botrytis* species in vitro. The selected resistant and susceptible lines were infected and screened to analyze the time series expression pattern of the genes after infection. RNA sequencing was conducted using onions collected at different time intervals after inoculation. Differentially expressed gene (DEG) analysis, variant analysis, and the Kyoto Encyclopedia of Genes and Genomes (KEGG) mapping were performed using RNA sequencing data. The results from RNA sequencing were validated by molecular biological experimental methods.

The results of this study expected to provide the information of gray mold resistance related genes in onion and SNPs validated by HRM analysis could be used as gene-related markers to screen for gray mold resistance traits in onion breeding systems.

## 2. Materials and Methods

### 2.1. Plant Material

In total, 54 onion lines used in onion breeding were obtained from Asia Seed Co., Ltd. (Seoul, Korea). For analyzing genetic relationships, seeds of 54 lines were sown in a seedling tray. Total genomic DNA (gDNA) was extracted from young leaves using a modified cetyl trimethyl ammonium bromide (CTAB) DNA extraction method [41]. The quality and concentration of the extracted genomic DNA stored at −20 °C were evaluated using a Nanodrop ND-1000 spectrophotometer (NanoDrop Technologies, Wilmington, DE, USA). To screen for gray-mold-resistant and susceptible onion lines under in vitro conditions, seeds of the 54 lines that were used for analyzing genetic relationships were sterilized and germinated in Murashige and Skoog (MS) basal medium [42]. Three onion leaves of each line were cut into 7–9 cm sections and transferred to new MS basal medium dispensed into square Petri dishes for infection with gray mold. The gray mold fungus that was used for infection was identified as *B. squamosa* using PCR [15]. A piece of *B. squamosa* was placed in 50 mL of Potato Dextrose Broth (PDB) and cultured in a shaking incubator (150 rpm, at 25 °C) for 4 days. In order to form conidia, it was cultured for 2 weeks in a shaking incubator (150 rpm, at 4 °C) under 315–400 nm UV-A and fluorescent conditions. After that, conidia were obtained by filtering the culture medium. Conidia infection was carried out by lightly pricking each onion leaf with a sterilized needle, and then injecting the pricked region with 1 µL (7.45 × 10^3^ cells/µL) of conidia with a pipette. The leaves were observed for lesions for five days (Figure S1). The size of the lesions was measured using ImageJ [43], and the obtained data were used to evaluate the resistance to gray mold. Evaluation was conducted in triplicate, and data were presented as the means with standard errors. Onion lines (Asia-23 and Asia-24) with the same genetic background that only differed in the gray mold resistance phenotype were selected and inoculated for analysis of time-series gene expression. The control plants were treated with distilled water. The leaves from the inoculated resistant onion line, susceptible line, and the control were collected at 0, 4, 8, 16, 24, and 32 h post inoculation (hpi) hereafter referred to as the resistant and susceptible time-series sample group, mock-treated control bulk, and stored at −75 °C until RNA sequencing.

### 2.2. RAPD-PCR and Analysis of Genetic Relationships

Genetic relationships of the 54 onion lines used in this study were analyzed by random amplified polymorphic DNA (RAPD)-PCR using 40 random primers (Operon Technologies, Alameda, CA, USA) and a PCR Pre Mix kit with a final reaction volume of 20 µL (*i-taq* Maximum PCR Pre Mix kit, iNtRON Biotechnology, Seongnam, Korea) containing 2.5-U i-Taq^TM^ *Taq* polymerase, 2.5 mM dNTPs, 1× reaction buffer with 10 µM primer, and 50 ng of gDNA. PCR was performed in a Veriti 96-Well Thermal Cycler (Applied Biosystems, Foster City, CA, USA) with the following program: initial denaturation at 95 °C for 10 min, 40 cycles of 1 min at 95 °C for denaturation, 1 min at 37 °C for annealing, 1 min at 72 °C for extension, and a final extension at 72 °C for 10 min. Amplified PCR products were analyzed by 1.5% agarose gel electrophoresis at 150 V, and polymorphic bands were viewed on an UV transilluminator. The RAPD-PCR analysis conducted using each primer set was prepared in triplicate to ensure reproducibility. The polymorphic amplicons obtained from the RAPD-PCR analysis were converted to binary data, depending on the presence of a polymorphic amplicon; the presence of a polymorphic amplicon was scored as 1, whereas its absence was scored as 0. A phylogenetic tree was constructed using these binary data and the UPGMA method based on Jaccard’s coefficient in XLSTAT (Addinsoft, Paris, France).

### 2.3. RNA Sequencing

For RNA isolation, leaf samples of the resistant and susceptible time-series sample groups and the mock-treated control bulk were pulverized in liquid nitrogen, and total RNA was extracted using an RNeasy Plant Mini kit (QIAGEN, Hilden, Germany). The integrity and quality of the RNA were evaluated using a Bioanalyzer RNA Chip (Agilent Technologies, Santa Clara, CA, USA). RNA library construction from each set of samples was performed using a TruSeq Stranded mRNA Sample Preparation kit (Illumina, San Diego, CA, USA) following the manufacturer’s instructions. The library size distribution and concentration were verified using the 2100 Bioanalyzer Desktop system (Agilent Technologies, Santa Clara, CA, USA). RNA sequencing was conducted using an Illumina Novadeq 6000 sequencer (Illumina, San Diego, CA, USA). The raw data obtained from RNA-seq were submitted to BioProject under the following ID number: PRJNA80361. For accurate analysis, pre-processing of RNA sequence raw data was performed using the sliding window (4), average quality (20), and minimum read size (50 bp) options in the Trimmomatic software [44]. Reference transcript sequence data for mapping of the RNA sequence data obtained in this study were constructed using data published by previous studies [27,45]. Reference data were pre-processed to reduce sequence redundancy and improve the performance of the analysis. The longest open reading frame (ORF) was extracted using the TransDecoder program embedded in the Trinity software [46], and sequences with protein evidence were selected through BLASTp and Pfam domain searches. Complete sequences were selected and clustered using the CD-HIT program [47]. Pre-processed RNA sequence data were mapped and aligned to pre-processed reference data, and then, the expected count of transcript expression level was calculated using HISAT2 [48] and StringTie [49]. Transcripts with expression levels similar to those of the mock-treated control were filtered.

### 2.4. Differential Gene Expression Analysis

The DEGs between samples were analyzed using DESeq [50] based on the read count of StringTie calculated at the transcript level. For DEG analysis, the read count was normalized using the size factor and dispersion. DEG analysis was performed using a false discovery rate (FDR) of less than 0.05, and a log_2_ fold change absolute value of 2 or more. Calculations for the resistant time-series sample group and susceptible time-series sample group were carried out by dividing each group into ten comparison groups. Normalized read count data of ‘Asia-23 0 hpi’ and ‘Asia-24 0 hpi’ were used as controls for comparison at each hpi tested within the resistant and susceptible sample groups, respectively. To avoid the log_2_ fold change value of each DEG comparison group being -inf (negative infinity) or + inf (positive infinity), the baseMean value, fold change value, and log_2_ fold change value for the two samples were calculated by adding 1 to the baseMean value of each sample. Based on this, an MA plot was executed for transcripts of each comparison group with baseMean > 1 for two samples in each comparison group. A volcano plot of transcripts was constructed for each comparison group using transcripts that satisfied the conditions used in the DEG analysis. Since the *Arabidopsis* genes have been analyzed for their functions, transcripts obtained through DEG analysis were matched to the Arabidopsis Information Resource (TAIR) records for identification, and matched transcripts were subjected to gene ontology (GO) annotation using the GO annotation tool [51]. For sequent DEG analysis at different times after gray mold inoculation in resistant and susceptible onion lines, normalized read count data in all hpi sections of Asia-23 and Asia-24 were converted to log_2_ fold scale, and the z-score was calculated. Hierarchical clustering was performed using the calculated z-score with one minus the Pearson correlation distance metric and the average linkage method in GENE-E (https://software.broadinstitute.org/GENE-E/, accessed on 10 December 2021). Clustered transcripts were matched to the TAIR records, and matched transcripts were conducted to GO annotation.

### 2.5. Analysis of Gene Network and Time-Series Expression

The processed transcripts were queried in TAIR, and the matched transcripts were mapped to the KEGG pathway database resource [52] to analyze the gene network related to gray mold resistance. Log normalization was performed to analyze the increase or decrease in the gene expression pattern with time after inoculation. Each gene expression level of the sample collected at 0 hpi was set to 0, and each gene expression level of the sample collected at 4, 8, 16, 24, and 32 hpi was log normalized to log_2_ (normalized read count of each hpi/normalized read count of 0 hpi).

### 2.6. RNA Extraction and Quantitative Real-Time PCR (qRT-PCR) Analysis

For quantitative real-time PCR (qPCR) analysis, total RNA of the resistant and susceptible time-series sample groups was extracted using a MiniBEST Plant RNA Extraction Kit (TaKaRa, Otsu, Japan) according to the manufacturer’s instructions. cDNA was synthesized from each sample using HiSenScript^TM^ RH [-] RT Pre Mix (iNtRON Biotechnology, Seongnam, Korea). The design of the primers for reverse transcription PCR (RT-PCR) and the qRT-PCR of the target genes were based on their transcript sequences obtained by RNA sequencing in this study. The actin gene, a housekeeping gene, was used as a control for qRT-PCR analysis. qRT-PCR analysis of target genes in each the resistant and susceptible time-series sample group was performed in an MIC thermocycler (BioMolecular Systems, Upper Coomera, Australia) using a 2× Real-Time PCR Master Mix kit with SFCgreen ^®^ I (Biofact, Seoul, Korea) under the following conditions: initial denaturation at 95 °C for 15 min, 45 cycles of 20 s at 95 °C, 30 s at 60 °C, 40 s at 72 °C; the specificity of amplicons was validated by the final melting curve stage from 65 °C to 95 °C. Fluorescence intensity data were collected at the end of each cycle using the instrument’s software. qRT-PCR analysis of each gene was conducted in triplicate, and the expression level of each gene was calculated from results of triplicate. Data were presented as the means with standard errors. Relative fold gene expression values of each sample were determined using the ΔCt method. The relative expression levels of samples at different time points (hpi) were calculated using the 2^−^^ΔΔ^^Ct^ method, which compared the ΔCt values of each sample (hpi) with the ΔCt values at 0 hpi [53].

### 2.7. Selected Genes Sequence Confirmation from gDNA of Asia-23 and Asia-24

The onion genome sequence data were used to confirm the sequences of *JAR1*, *COI1*, and *MYC2* genes selected through gray-mold-resistance-related gene screening using RNA sequencing data from gDNA of Asia-23 and Asia-24. Gene annotation was performed by local BLAST to onion genome sequence data using the CDS sequences of *JAR1*, *COI1*, and *MYC2* obtained in this study [54]. Based on the annotation data, PCR primers (Table S1) were designed to target and amplify the start codon, exons, and stop codons of each gene. PCR was performed using a BioFACT^TM^ H-start Taq PCR Pre Mix kit (Seoul, Korea), Asia-23 and Asia-24 gDNA, and designed primer sets. The PCR program was as follows: initial denaturation at 95 °C for 15 min, 35 cycles of 20 s at 95 °C, 30 s at the annealing temperature of each primer set, 20 s at 72 °C, and 5 min at 72 °C. Each PCR product was subjected to electrophoresis to confirm the target amplicon, eluted using a P&C Multiple Elution Kit (Suwon, Korea), and the elution products were sequenced using Celemics (Seoul, Korea) BTSeq^TM^ Contiguous Sequencing service.

### 2.8. High Resolution Melting Analysis

The primer set for high resolution melting (HRM) analysis was designed using the Primer3 software [55] (Table S2). The HRM analysis was conducted in triplicate using an MIC thermocycler (BioMolecular Systems, Upper Coomera, Australia) and a 2× Real-Time PCR Master Mix kit with SFCgreen ^®^ I (Biofact, Seoul, Korea). The final reaction contained 0.75 µM of each primer pair, and 10 ng of the resistant and susceptible onion template gDNA in a 10 µL reaction volume. The HRM analysis programs were as follows: initial denaturation at 95 °C for 15 min, 45 cycles of 20 s at 95 °C, 30 s at 60 °C, and 20 s at 72 °C. Then, the HRM analysis was conducted using an initial hold at 65 °C for 1 min, and ramping from 65 °C to 95 °C in 0.1 °C steps. Fluorescence signal data were acquired after 2 s of each step. The HRM data from each replicate were analyzed using the MIC thermocycler software (BioMolecular Systems, Upper Coomera, Australia) and the DNA melting peak profile-based HRM (MP-HRM) analysis method that was previously reported [56].

## 3. Results

### 3.1. Screening Gray-Mold-Resistant and Susceptible Onions

**Figure 1:**
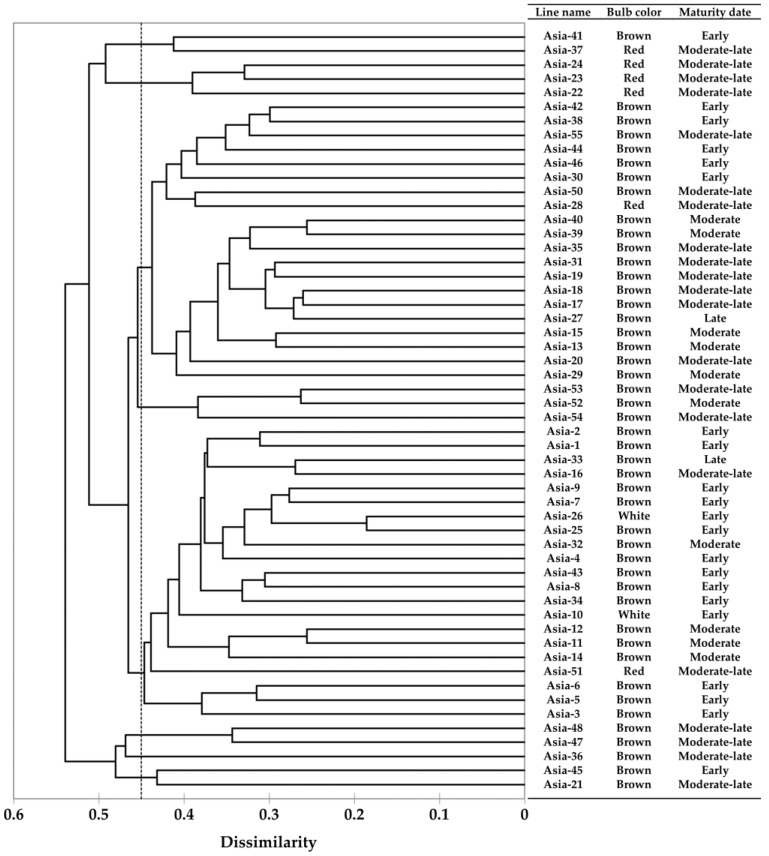
Dendrogram of 54 onion lines provided by Asia seed Co., Ltd. (Seoul, Korea) by RAPD analysis and information on the bulb color and maturity date of each line.

To analyze the expression pattern of gray mold resistance-related genes, screening was carried out to select onion lines with the same genetic background that only differed in the gray mold resistance phenotype. Genetic relationships among the onion lines were determined by RAPD-PCR, and 148 polymorphic amplicons were obtained. The dendrogram constructed from the polymorphic amplicon data showed a genetic similarity of 82.08% within the groups and 17.97% between the groups (Figure 1).

**Figure 2:**
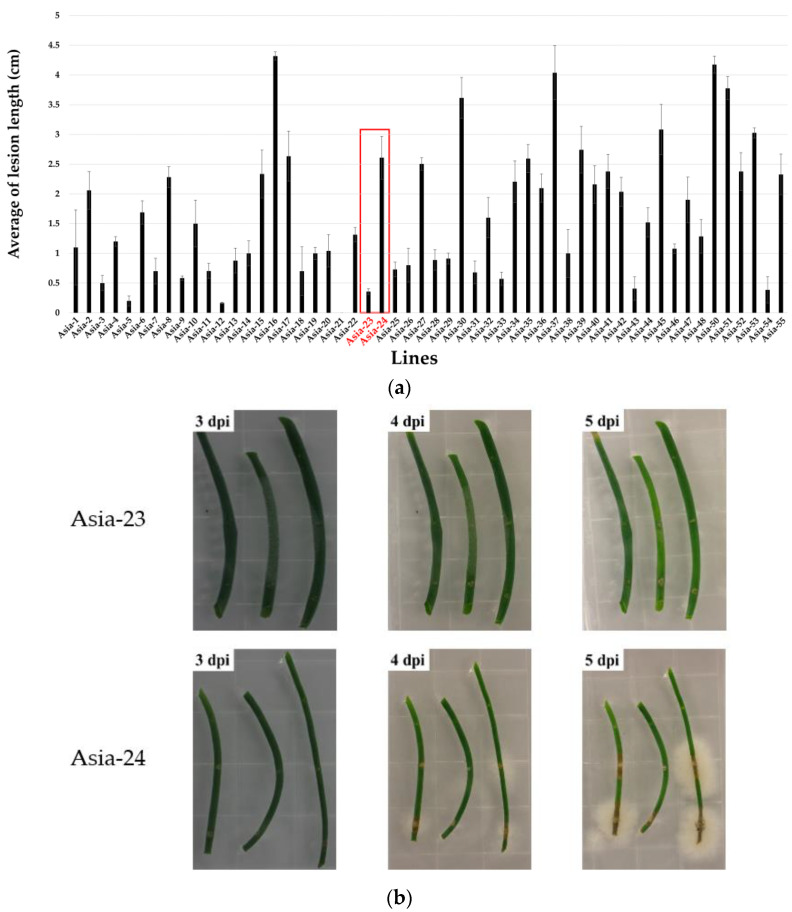
Results of screening gray-mold-resistant and susceptible onions. (**a**) Evaluation of lesion length on 54 onion lines inoculated in vitro. Error bars indicate the standard error of three biological replicates. (**b**) In vitro inoculation results. Asia-23 and Asia-24 in red color showed gray mold resistance and susceptible phenotype. A representative image of lesion appearance in inoculated leaves was taken at 3–5 days post inoculation.

The onion lines that showed a weak disease incidence, measured by the length of the lesion, were Asia-5, Asia-9, Asia-12, Asia-21, Asia-23, and Asia-53. The onion lines with a strong disease susceptibility were Asia-15, Asia-16, Asia-24, Asia-49, Asia-50, and Asia-52 (Figure 2a). Based on the genetic relationships of the onion lines and the lesion measurements after inoculation with *B. squamosa*, the lines Asia-23 and Asia-24, which showed most pronounced differences for gray mold resistance and susceptibility, were selected (Figure 2b). The temporal expression pattern of gray-mold-resistance-related genes in Asia-23 (gray mold resistant), and Asia-24 (gray mold susceptible) was assessed by inoculating the lines with gray mold in vitro. Onion leaf samples of these two lines were collected at 0, 4, 8, 16, 24, and 32 hpi.

### 3.2. RNA Sequencing and Sequence Data Pre-Processing

Quality assessment of the total RNA based on the A260/280 and A260/230 ratios, indicators of RNA integrity and quality [57], showed that the RNA was suitable for subsequent analyses. The 28S/18S ratio indicated that the RNA was intact. The RNA integrity number (RIN) value [58] was approximately 6 in samples, which was considered satisfactory for subsequent analyses according to the previously reported RIN standard [59]. The number of total read bases was between 5.1 and 5.4 billion, and the total number of reads was between 50 million and 54 million. More than 93% of bases in RNA sequences were with a Phred quality score equal to or greater than 30 for all samples (Table S3). Therefore, RNA sequencing was confirmed to be good for subsequent analysis. After pre-processing, the total number of read bases was between 4.5 billion and 4.9 billion, and the total number of reads was from approximately 45 to 49 million. Approximately 8–12% of reads and bases were trimmed from the raw RNA sequence data (Table S4). Clustering of the selected sequences among the reference sequences using the CD-HIT program reduced the existing 100,926 transcript sequences to 42,921 sequences. These sequences were used as reference data for subsequent analysis. After the alignment of the pre-processed RNA sequences to the reference data, 40,447 transcript sequences were mapped to the 42,921 reference transcript sequences, with a mapping rate from 74.3% to 85.9% and an average mapping rate of 81% (Table S5).

### 3.3. Differential Gene Expression Analysis

**Figure 3:**
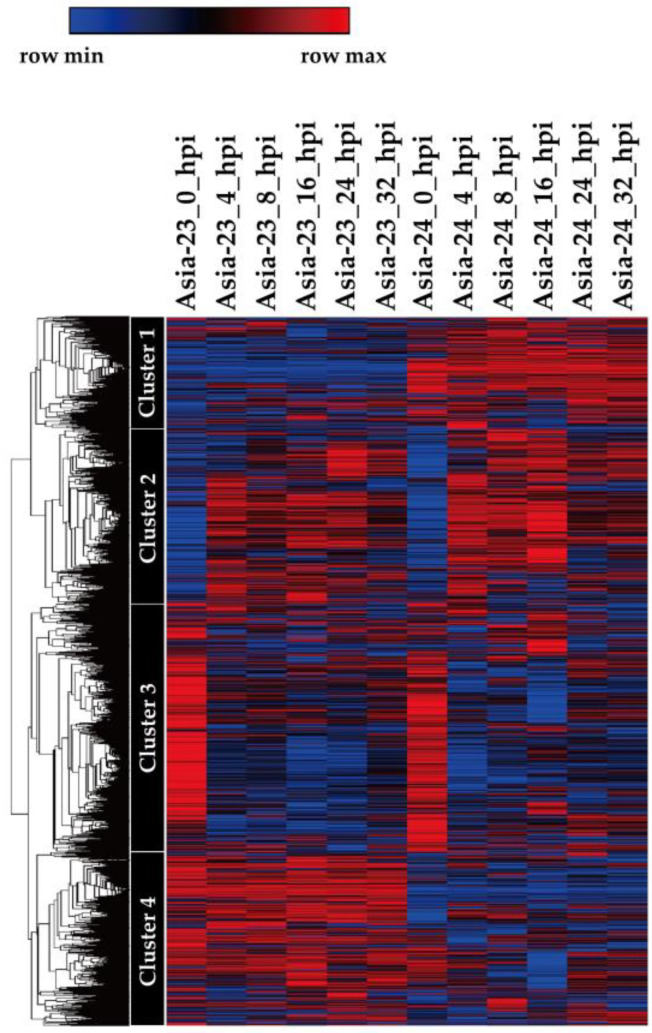
Hierarchical clustering analysis for time-series gene expression in Asia-23 and Asia-24. Cluster 1: Transcripts showed that the expression level in Asia-23 was lower than that in Asia-24 at all hpi points, Cluster 2: Transcripts with increased expression levels after 0 hpi in Asia-23 and Asia-24, Cluster 3: Transcripts with decreased expression levels after 0 hpi in Asia-23 and Asia-24, Cluster 4: Transcripts showed that the expression level in Asia-24 was lower than that in Asia-23 at all hpi points. The red and blue coloring indicates relatively high and low expression levels, respectively, as displayed in the scale bar.

The gene expression at each hpi was calculated using the gene expression at 0 hpi as a control. At all time points, the number of upregulated transcripts was greater than the number of downregulated transcripts in Asia-23 and Asia-24 (Table S6). Upregulated transcripts and downregulated transcripts were matched to TAIR resource, and the matched transcripts were conducted GO annotation. Transcripts matched to the ‘response to stress’ and ‘response to biotic stimulus’ of the ‘GO Biological process’, which were considered to relate with gray mold resistance trait were selected (Supplementary File S1). 194 and 186 transcripts were annotated among the upregulated transcripts in Asia-23 and Asia-24 at 4 hpi, respectively. In upregulated transcripts at 8 hpi, 175 transcripts from Asia-23 were annotated and 159 transcripts from Asia-24 were annotated. In the case of downregulated transcripts at 4 hpi, 22 transcripts from Asia-23 and 41 transcripts from Asia-24 were annotated, and at 8 hip, 21 and 39 downregulated transcripts from Asia-23 and Asia-24 were annotated, respectively. The DEG results were expressed as MA and volcano plots (Figures S2 and S3). The MA and volcano plots confirmed that the number of upregulated genes after inoculation was significantly higher than the number of downregulated genes. After hierarchical clustering, a total of 40,447 transcripts were clustered into four groups (Figure 3).

The first cluster with 6403 transcripts showed that the expression level in Asia-23 was lower than that in Asia-24 at all hpi points. The second cluster consisted of 9890 transcripts with increased expression levels after 0 hpi in Asia-23 and Asia-24. The third cluster consisted of 14,254 transcripts with decreased expression levels after 0 hpi in Asia-23 and Asia-24. The fourth cluster had 9900 transcripts showing higher expression levels for Asia-23 than for Asia-24 in all hpi. Of the 9900 transcripts in Cluster 4, 4317 transcripts matched those in TAIR. The matched transcripts were assigned to various GO sub-categories of ’GO Cellular Component,’ ’GO Molecular Function,’ and ‘GO Biological process’ (Figure S4). Among them, 1048 and 439 transcripts were annotated to ‘response to stress’ and ‘response to biotic stimulus,’, respectively, of the ‘GO Biological process,’ suggesting that they are related to the gray mold resistance trait.

### 3.4. Analysis of Gene Network and Time-Series Expression

**Figure 4:**
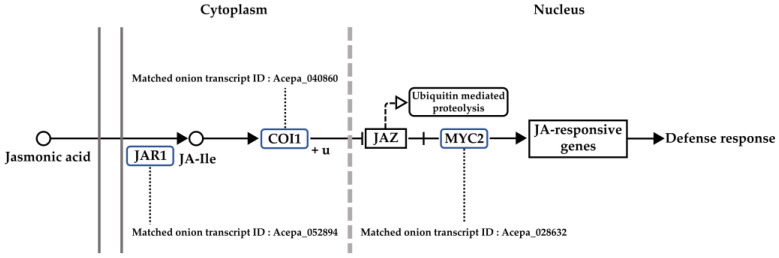
Matched onion transcripts to genes involved in the ‘plant hormone signal transduction: α-linolenic acid metabolism’ pathway. *JAR1*: *Jasmonate-resistant 1*, *COI1*: *Coronatine-insensitive protein 1*, *MYC2*: *transcription factor MYC2*.

KEGG pathway analysis of the 40,447 onion transcripts expressed after gray mold inoculation matched 11,726 transcripts to TAIR records and inferred their function. After the KEGG pathway analysis of the 11,726 matched onion transcripts, ‘α-linolenic acid metabolism’, ‘MAPK signaling pathway’, ‘Plant hormone signal transduction’, and ‘Plant-pathogen interaction’, which are considered to be related to gray mold resistance, were selected (Figure S5 and Supplementary File S2). The analysis of the time-series gene expression pattern after inoculation with gray mold of each annotated gene revealed that the expression pattern of transcripts matched with the *JAR1* (AT2G46370), *COI1* (AT2G39940), and *MYC2* (AT1G32640) genes in the ‘plant hormone signal transduction: α-linolenic acid metabolism’ pathway (Figure 4) were considered significantly related to gray mold resistance trait.

### 3.5. Verification of Gene Expression Using qRT-PCR

**Figure 5:**
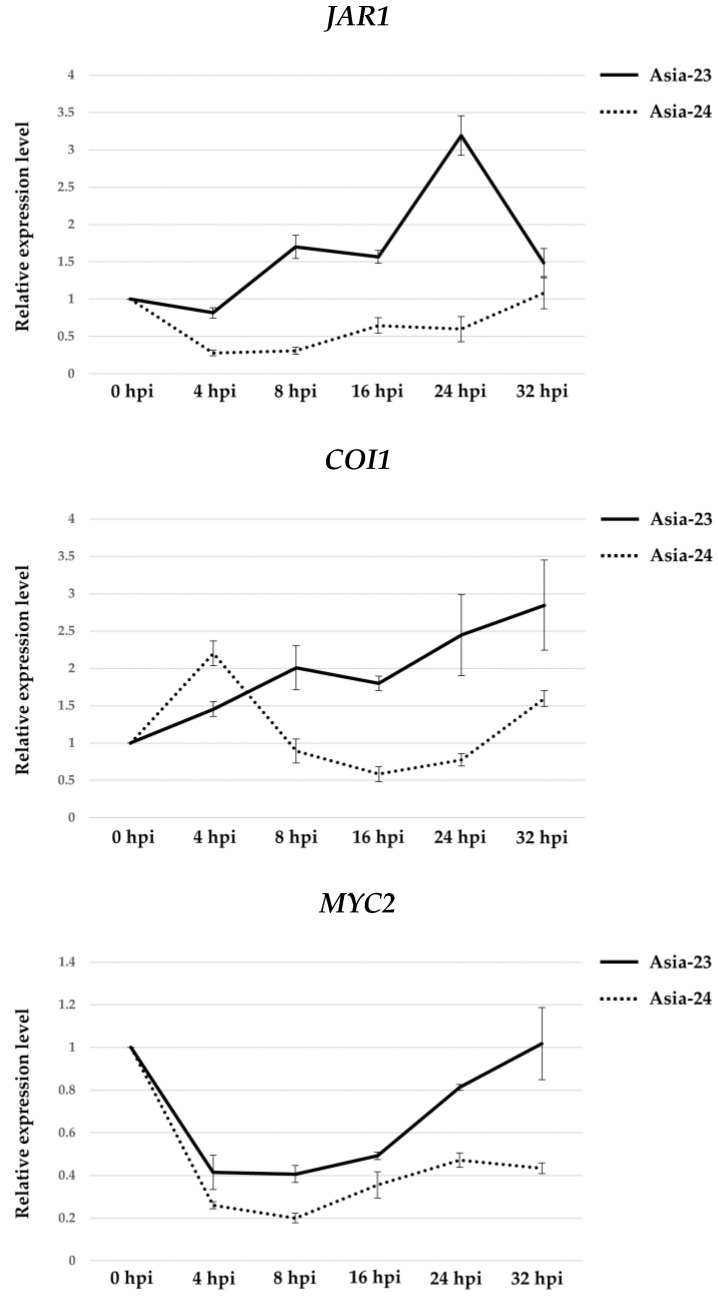
Comparison of time-series gene expression pattern of each selected gene in Asia-23 and Asia-24 by qRT-PCR. The solid line shows the time-series gene expression pattern of Asia-23, the dotted line represents the time-series gene expression of Asia-24. The relative expression levels of samples at different time points (hpi) were calculated using the 2^−ΔΔCt^ method, which compared the ΔCt values of each sample (hpi) with the ΔCt values at 0 hpi. Error bars indicate the standard deviation of three biological replicates. Hpi: hours post inoculation.

qRT-PCR was performed to confirm that the gene expression patterns of *JAR1*, *COI1,* and *MYC2* belong to the ‘plant hormone signal transduction: α-linolenic acid metabolism’ pathway (Figure 5). In Asia-23, the expression of *JAR1* decreased slightly at 4 hpi, but increased 1.70-fold at 8 hpi. At 24 hpi, the expression increased 3.19-fold, and at 32 hpi, the expression decreased slightly. In Aisa-24, the expression of *JAR1* decreased 0.28-fold at 4 hpi, and then gradually increased up to 1.07-fold at 32 hpi when compared with that at 0 hpi. The expression of *COI1* gradually increased after 0 hpi in Asia-23, up to 2.84-fold at 32 hpi. In Asia-24, the expression temporarily increased 2.20-fold at 4 hpi, but at 16 hpi, it gradually decreased to 0.58-fold its level at 0 hpi, followed by a gradual increase thereafter. The expression level of *MYC2* decreased in both Asia-23 and Asia-24 at 4 hpi. However, in Asia-23, the decrease in expression was smaller than that in Asia-24 and gradually increased after 4 hpi until almost reaching the initial expression levels at 32 hpi. In Asia-24, the expression decreased up to 0.20-fold by 8 hpi and gradually recovered, although at 32 hpi, the expression level was 0.43-fold lower than that at 0 hpi.

### 3.6. Confirmation of Selected Gene Sequences from gDNA of Asia-23 and Asia-24 and HRM Analysis

**Figure 6:**
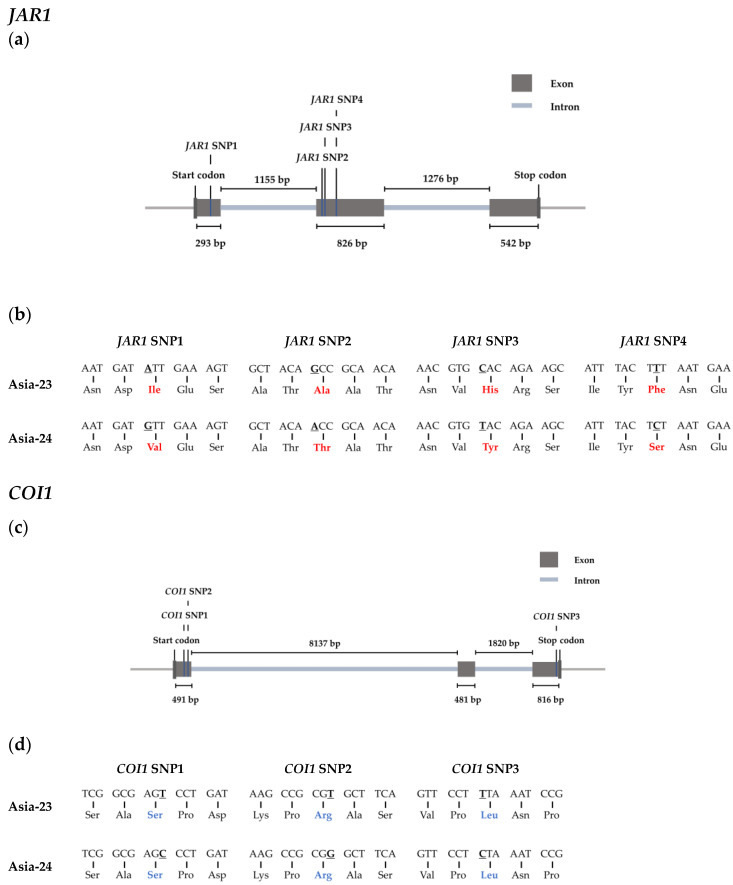

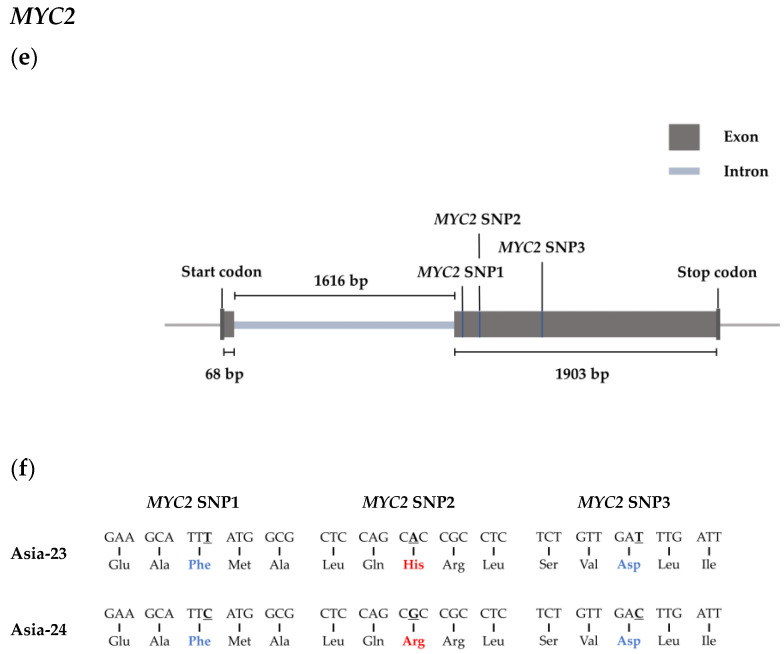
Confirmation of selected gene sequences from gDNA. (**a**) Structure of *JAR1* gene and the information of each region. (**b**) SNPs and amino acid sequences of *JAR1* gene between Asia-23 and Asia-24. (**c**) Structure of *COI1* gene and the information of each region. (**d**) SNPs and amino acid sequences of *COI1* gene between Asia-23 and Asia-24. (**e**) Structure of *MYC2* gene and the information of each region. (**f**) SNPs and amino acid sequences of *MYC2* gene between Asia-23 and Asia-24. Bold and underlined: SNP, Red: Synonymous mutations, Blue: Non-synonymous mutations.

In Asia-23 and Asia-24, the start codon, exons, and stop codon sequences of *JAR1*, *COI1*, and *MYC2* were confirmed using the onion genome sequence data (Figure 6). The length from the start codon to the stop codon of *JAR1*, *COI1*, and *MYC2* was confirmed to be 4101 bp, 11,745 bp, and 3587 bp, respectively. The *JAR1* and *COI1* genes consisted of three exons and two introns, and *MYC2* consisted of two exons and one intron. An exonic single nucleotide polymorphism (SNP) existed between Asia-23 and Asia-24. Four non-synonymous SNPs were identified in the exon of *JAR1*, and three synonymous SNPs were present in the exon of *COI1*. Three SNPs were present in the exon of *MYC2*, one of which was non-synonymous, and the other two were synonymous (Figure 6).

**Figure 7:**
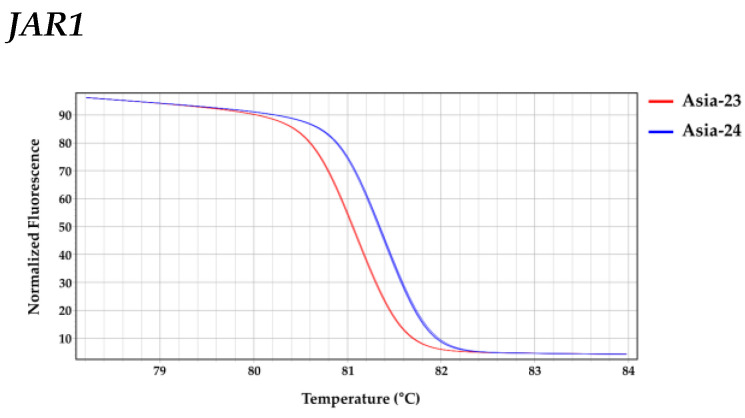

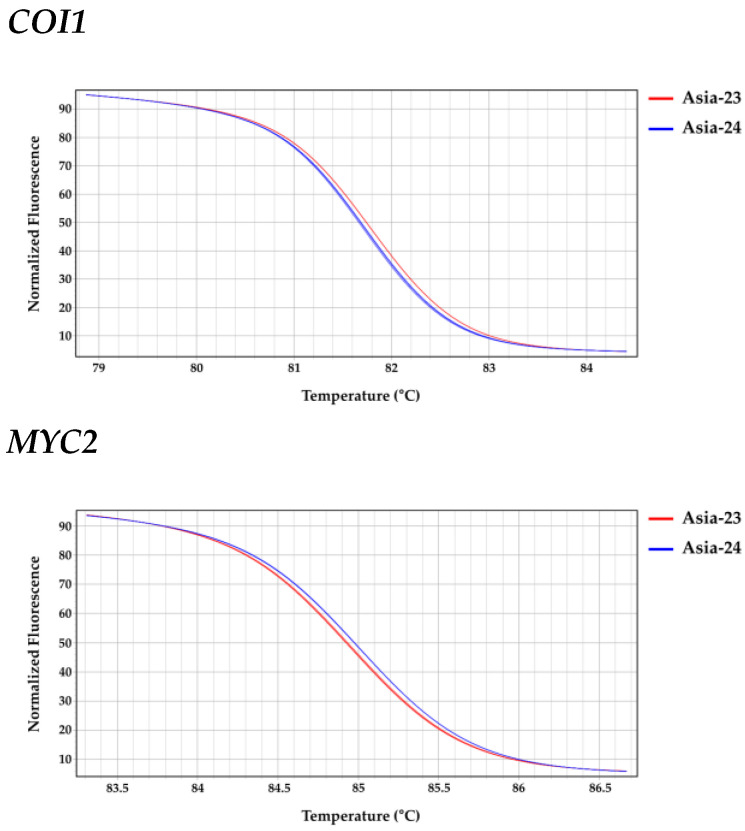
HRM analysis of each selected gene in Asia-23 and Asia-24. Red line: Average normalized fluorescence curve of Asia-23, Blue line: Average normalized fluorescence curve of Asia-24.

After selecting one of the SNPs present in each gene, HRM analysis was performed by targeting the SNPs. The HRM triplicate data of each gene showed that the least squares mean (LS-mean) for the melting peak temperature of *JAR1* amplicons was 81.387 °C for Asia-23 and 81.730 °C for Asia-24; 81.783 °C for Asia-23 and 81.730 °C for Asia-24 for *COI1*; and 85.007 °C for Asia-23 and 84.953 °C for Asia-24 for *MYC2* (Figure 7 and Table 1). HRM analysis of each gene using the MP-HRM analysis method statistically confirmed the sequence polymorphism of each gene between Asia-23 and Asia-24.

## 4. Discussion

Screening disease-resistant breeding lines and understanding genetic mechanisms involved in disease resistance traits are important for reducing crop losses caused by diseases and for sustainable agriculture. The dendrogram based on the genetic relationship of 54 lines showed that lines with similar bulb color or maturity date were clustered together (Figure 1). From the inoculation experiments, two lines showing gray-mold-resistant and susceptible phenotype were selected: Asia-23 resistant to gray mold and Asia-24 susceptible to gray mold.

The DEG analysis confirmed that the ratio of genes whose expression increased 4-fold or more after 0 hpi was high at all sampled time points in Asia-23 and Asia-24. This pattern is similar to that reported for gene expression in other crops inoculated with gray mold [60,61]. Early defense against pathogens in plants is important. To this end, plants detect the invasion of pathogens by recognizing pathogen patterns, and activate resistance related genes through rapid signal transmission to perform various resistance mechanisms. Although the initial penetration stage of *Botrytis* varies according to the target plant host, it was from 4 to 12 h after infection [62]. When *botrytis* was inoculated into gray-mold-resistant grapevine, antioxidative activities were increased up to 8 hpi, and subsequent infection progresses by gray mold triggered sustained reactive oxygen species production did not occur [63]. In addition, it was reported that the defense gene expression was higher in gray-mold-resistant tomatoes than in susceptible tomatoes at 8 hpi [64]. The results of this study showed that the number of upregulated transcripts annotated to ‘response to stress’ and ‘response to biotic stimulus’ at 4 hpi and 8 hpi of Asia-23 were greater than Asia-24. However, downregulated transcripts of Asia-24 at 4 hpi and 8hpi were greater than Asia-23 and these results were presumed to be related to the gray mold resistance trait of Asia-23. As a result of hierarchical clustering analysis, it was considered that the genes belonging to Cluster 4, in which a high expression in Asia-23 during all hpi points was observed, were related to gray mold resistance in onion. Among the 4317 transcripts matched to TAIR in Cluster 4, 1048 were annotated to ‘response to stress’ and 439 to ‘response to biotic stimulus.’ The transcripts with a higher expression in Asia-23 than in Aisa-24 and those annotated to ’response to stress’ and ‘response to biotic stimulus’ are likely related to the gray mold resistance trait of Asia-23.

The TAIR-matched transcripts were mapped to the KEGG pathways, and those associated with gray mold resistance were selected. *JAR1*, which is involved in the JA signaling pathway, encodes a jasmonate-amido synthetase that catalyzes the formation of a biologically active jasmonoyl-isoleucine (JA-Ile) conjugate that induces plant defense and pathogen resistance [65,66,67]. The *COI1* gene encodes the F-box protein, a component of the E3 ubiquitin ligase [68]. COI1 forms a co-receptor complex with jasmonate zinc-finger inflorescence meristem (ZIM)-domain (JAZ) protein, which acts as a repressor in the JA signaling pathway, and bioactive JA-Ile binds to the high-affinity co-receptor [69]. After that, JAZ proteins are moved to the 26 s proteasome and degraded, and transcription factors, such as MYC2 that activate the expression of downstream JA-responsive genes, are released [38].

The expression pattern of *JAR1* validated by qRT-PCR, gradually increased in Asia-23 (resistant line) and decreased in Asia-24 (susceptible line). In previous studies, susceptibility to *B. cinerea* was increased when inoculated into *jar1* mutant of *Arabidopsis* which was suppressed the expression of *JAR1* [70]. Based on previous studies and the decreased expression pattern of *JAR1* gene in Asia-24 suggesting that the expression of the *JAR1* gene was significantly associated with gray mold resistance.

The expression of *COI1* temporarily increased at 4 hpi in Aisa-24, and then decreased sharply by 16 hpi. Its expression levels after 24 and 32 hpi were lower than those in the Asia-23 line. In contrast, the expression continued to increase in Asia-23 after inoculation. In *Arabidopsis*, susceptibility to *Alternaria brassicicola* (Schwein.) Wiltshire, *Botrytis cinerea*, *Plectosphaerella cucumerina* (Lindford) W. Gams, and *Fusarium oxysporum* Schlechtend. negatively correlated with *COI1* expression [71,72,73]. The reason for the transient high expression of *COI1* at 4 hpi in the Asia-24 line is unclear. However, given that the expression was lower than that in the Asia-23 line at all subsequent time points, it could be presumed that the *COI1* gene is related to the gray mold resistance trait. The expression of *MYC2* decreased at 4 hpi in both the Asia-23 and Asia-24 lines. However, in the Asia-23 line, the expression gradually increased after 4 hpi, whereas in the Asia-24 line, expression decreased until 8 hpi, followed by a slight increase after 8 hpi. The expression of *MYC2* in *Arabidopsis* negatively affects resistance to necrotrophic pathogens [74]. In contrast, in tomato (*Solanum lycopersicum* L.), susceptibility was increased when *B. cinerea* was inoculated into *MYC2-RNAi* plants with a reduced expression of *MYC2* [75]. The expression pattern of *MYC2*, in the present study, was not completely consistent with the expression pattern of *MYC2* in other crops. However, because the expression at 4 hpi was reduced in both lines, and the expression level in the Asia-23 line was higher at all time points when compared with that in the Asia-24 line, the expression of the *MYC2* gene was considered to have positive effects on gray mold resistance in onions, similar to tomatoes.

SNPs were found in the *JAR1*, *COI1*, and *MYC2* sequences between the Asia-23 and Asia-24 lines. Amino acid substitution by the non-synonymous SNP was confirmed in *JAR1* and *MYC2*. It was considered that the amino acid substitution affected the structure or function of the corresponding protein related to gray mold resistance, as reported in other studies [76,77]. Only synonymous SNPs were present in the *COI1* sequences. However, synonymous SNPs are also known to affect mRNA secondary structure, stability, and translation rate [78]. Therefore, it could be presumed that the function of the *COI1* gene was influenced by the synonymous SNP that affected the gray mold resistance trait.

Molecular markers, such as restriction fragment length polymorphisms [79], RAPD [80], amplified fragment length polymorphisms [81], simple sequence repeats, and SNPs [82,83,84] have been developed in onion for a variety of purposes. However, molecular markers for screening disease resistance traits are still lacking. The SNPs in each gene were validated by HRM analysis, and these SNPs could be used as gene-related markers to screen for gray mold resistance traits in onion breeding systems.

## 5. Conclusions

The degree of resistance to gray mold disease of 54 onion lines was evaluated using an onion inoculation system developed in this study. Subsequently, gray-mold-resistant onion line ‘Asia-23’ and gray mold susceptible onion line ‘Asia-24’ were selected. The two lines had a close genetic relationship with the highest differences in gray mold resistance. Both lines were subjected to in vitro inoculation to analyze the time-series gene expression patterns after gray mold inoculation. The samples were obtained at 0, 4, 8, 16, 24, and 32 h after gray mold inoculation. RNA sequencing data were utilized for DEG analysis, GO annotation, and KEGG mapping. Time-series gene expression patterns of transcripts that matched the *JAR1* gene involved in the jasmonic acid signaling pathway and known to be related to gray mold resistance were compared between the Asia-23 and Asia-24 lines. The expression patterns of both lines were validated by qRT-PCR. Analysis of the expression patterns of *COI1* and *MYC2* genes, which are similarly to *JAR1* and involved in the jasmonic acid signaling pathway, confirmed that the *JAR1*, *COI1*, and *MYC2* genes were significantly related to gray mold resistance in onion. SNPs that existed between the Asia-23 and Asia-24 lines in the *JAR1*, *COI1*, and *MYC2* gene sequences were validated by HRM analysis. These results are expected to be useful for subsequent studies on genes associated with disease resistance in onions and the breeding of onions with gray mold resistance traits.

## Figures and Tables

**Table 1:** HRM analysis results of each gene statistically grouped using the MP-HRM analysis method.

Gene	Line	LS Means (Melting Peak Tm (°C))	Groups *	
*JAR1*	Asia-24	81.387	A	
	Asia-23	81.093		B
*COI1*	Asia-23	81.783	A	
	Asia-24	81.730		B
*MYC2*	Asia-24	85.007	A	
	Asia-23	84.953		B

* In each column: means with the same letter are not significantly different.

## Data Availability

The raw data of RNA-seq have been deposited with links to BioProject accession number “PRJNA803261” in the NCBI.

## Supplementary Materials

The following Supplementary Materials are available online at https://www.mdpi.com/article/10.3390/genes13030542/s1, Figure S1: In vitro inoculation for screening gray-mold-resistant and susceptible onions using detached onion leaves and conidia of *B. squamosa*. Figure S2: Differentially expressed gene (DEG) results expressed as MA plot. Figure S3: Differentially expressed gene (DEG) results expressed as Volcano plot. Figure S4: GO annotation of transcripts clustered in Cluster 4. Figure S5: Results of KEGG pathway analysis considered to be related to gray mold resistance. Table S1: Primer sets for confirmation of selected gene sequences. Table S2: Primer sets for high-resolution melting (HRM) analysis. Table S3: Statistics of sequence raw data. Table S4: Pre-processing results of onion RNA sequence raw data. Table S5: Results of mapping and alignment to reference data of pre-processed RNA sequence data. Table S6: DEG comparison at each hour post inoculation (hpi) within the resistant and susceptible sample groups. File S1: Identification of DEGs in Asia-23 and Asia-24. File S2: A list of onion transcripts matched to the KEGG map.
