# Supplementary material for: Using Transcriptome Analysis to Explore Gray Mold Resistance-Related Genes in Onion (Allium cepa L.)
Source: Genes (Basel). 2022 Mar 18;13(3):542. doi: 10.3390/genes13030542 (PMC8955018; doi:10.3390/genes13030542)
Supplement: Supplementary file 1 [file genes-13-00542-s001.zip › (Proofreading) Supplementary materials.pdf]

Supplementary materials

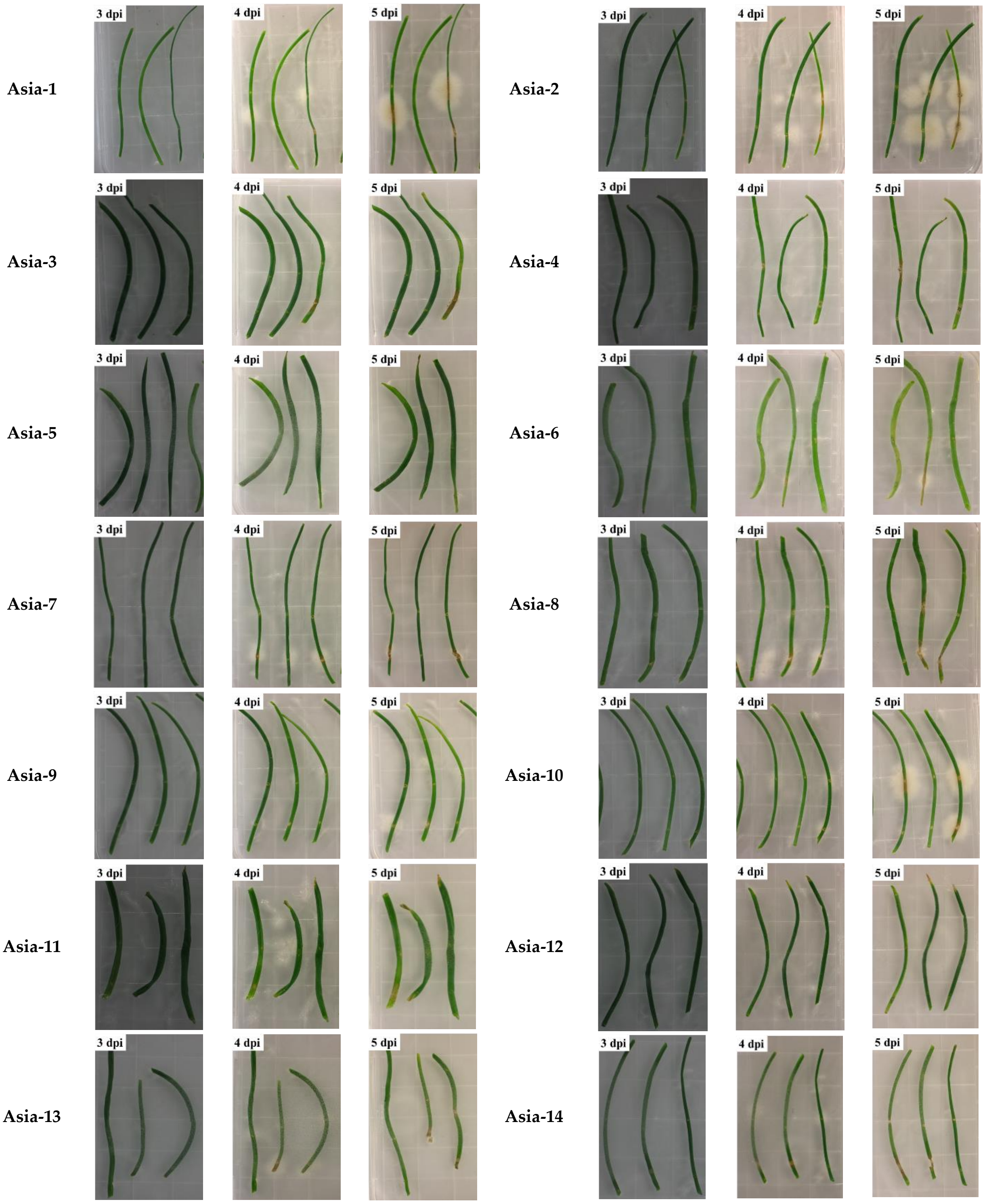

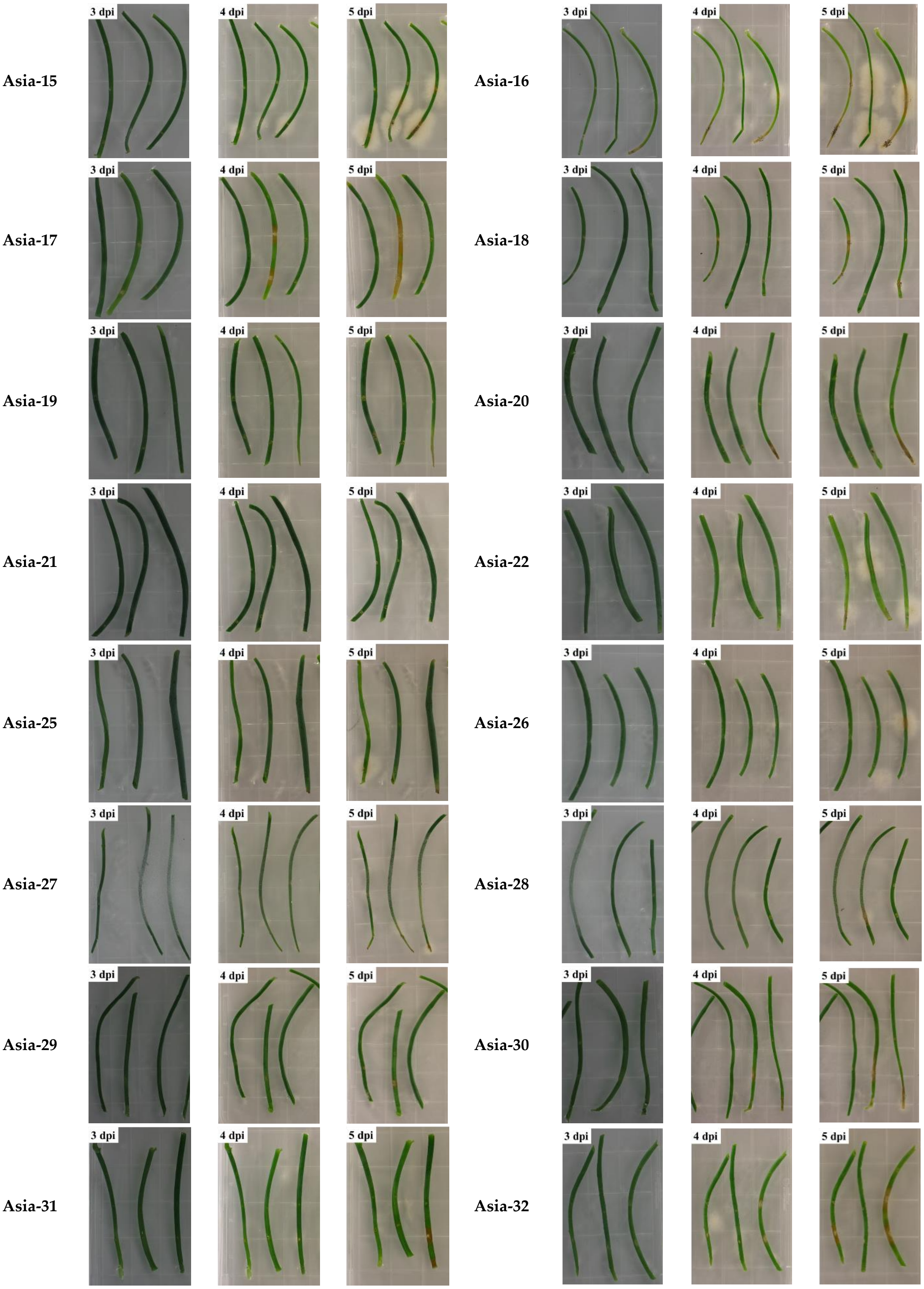

Asia-15
3 dpi
4 dpi
5 dpi
Asia-16
Asia-17
Asia-18
Asia-19
Asia-20
Asia-21
Asia-22
Asia-25
Asia-26
Asia-27
Asia-28
Asia-29
Asia-30
Asia-31
Asia-32

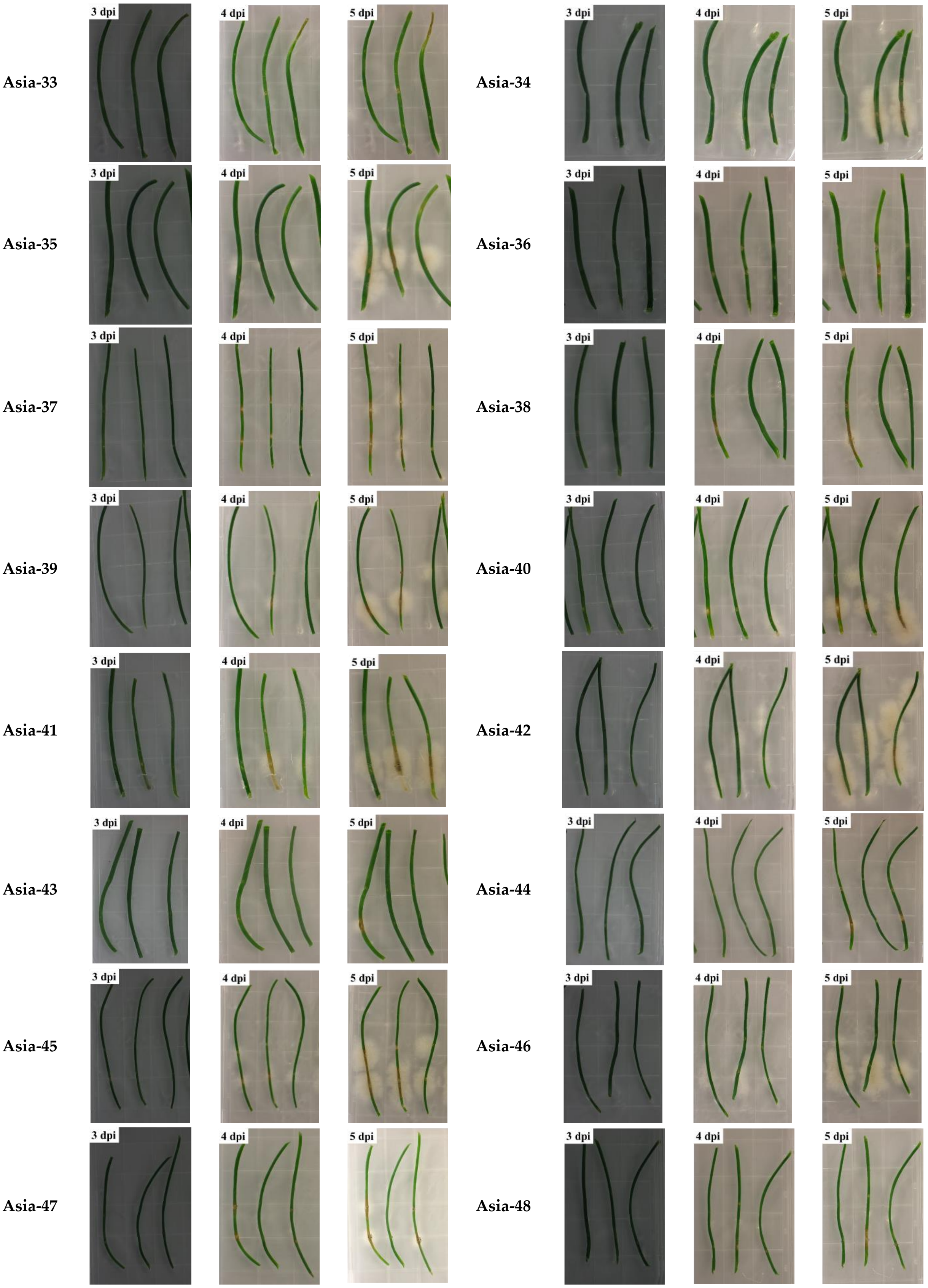
Asia-33
3 dpi
4 dpi
5 dpi
Asia-34
3 dpi
4 dpi
5 dpi
Asia-35
3 dpi
4 dpi
5 dpi
Asia-36
3 dpi
4 dpi
5 dpi
Asia-37
3 dpi
4 dpi
5 dpi
Asia-38
3 dpi
4 dpi
5 dpi
Asia-39
3 dpi
4 dpi
5 dpi
Asia-40
3 dpi
4 dpi
5 dpi
Asia-41
3 dpi
4 dpi
5 dpi
Asia-42
4 dpi
5 dpi
Asia-43
3 dpi
4 dpi
5 dpi
Asia-44
3 dpi
4 dpi
5 dpi
Asia-45
3 dpi
4 dpi
5 dpi
Asia-46
3 dpi
4 dpi
5 dpi
Asia-47
3 dpi
4 dpi
5 dpi
Asia-48
3 dpi
4 dpi
5 dpi

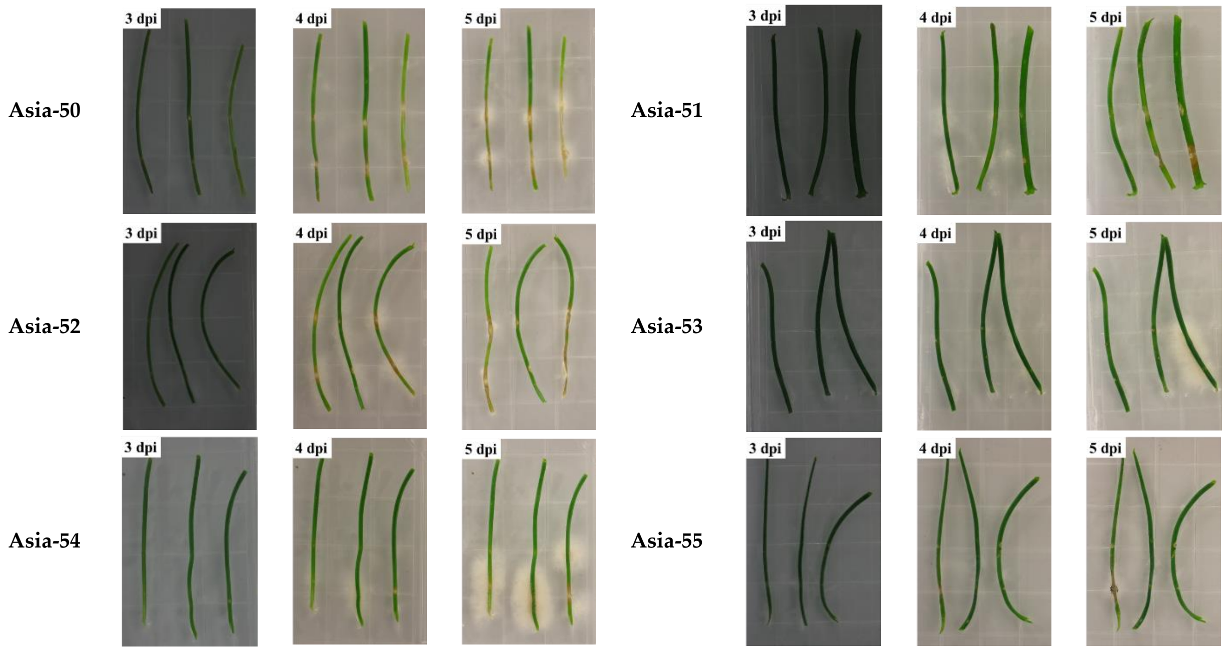

Figure S1. *In vitro* inoculation for screening gray mold resistant and susceptible onion lines using detached onion leaves and conidia of *B. squamosa*. A representative image of lesion appearance in inoculated leaves were taken at 3 – 5 days post inoculation (dpi).

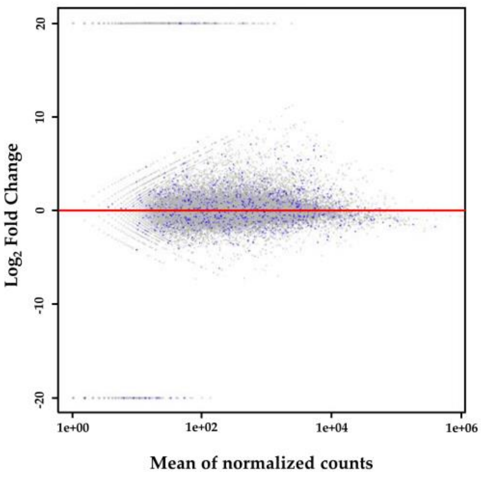

Asia-23 0 hpi vs 4 hpi

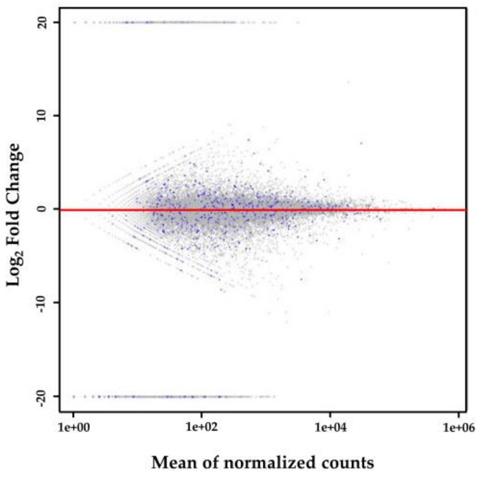

Asia-24 0 hpi vs 4 hpi

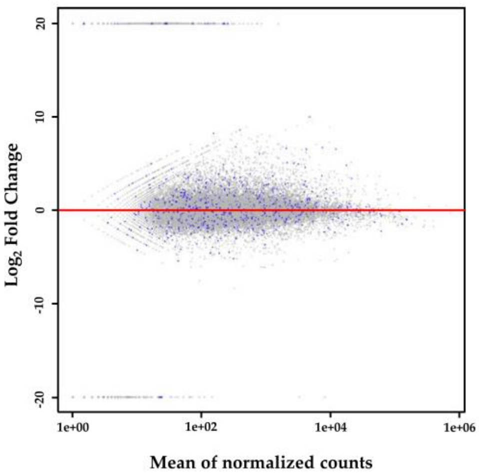

Asia-23 0 hpi vs 8 hpi

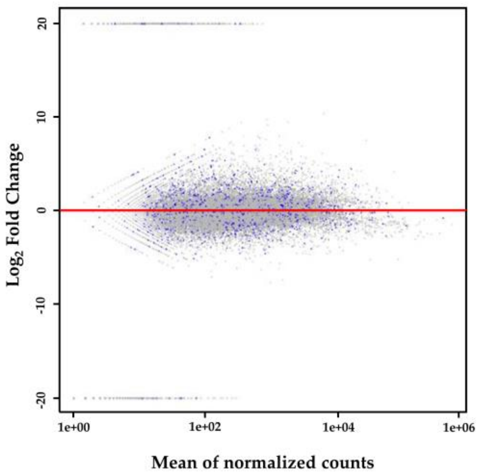

Asia-24 0 hpi vs 8 hpi

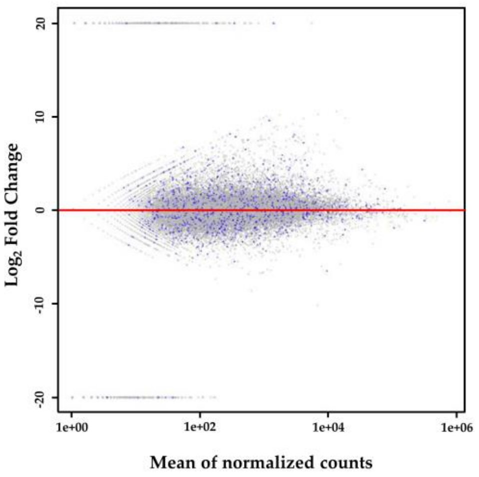

Asia-23 0 hpi vs 16 hpi

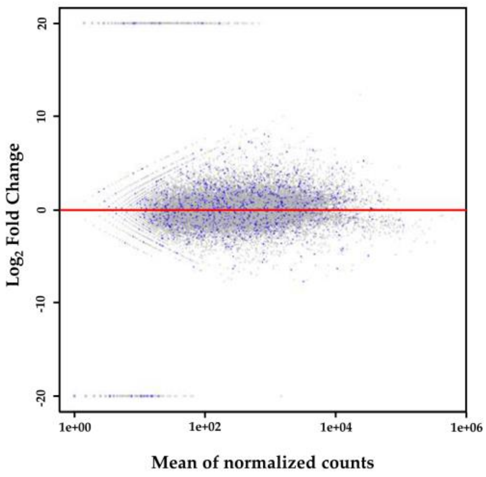

Asia-24 0 hpi vs 16 hpi

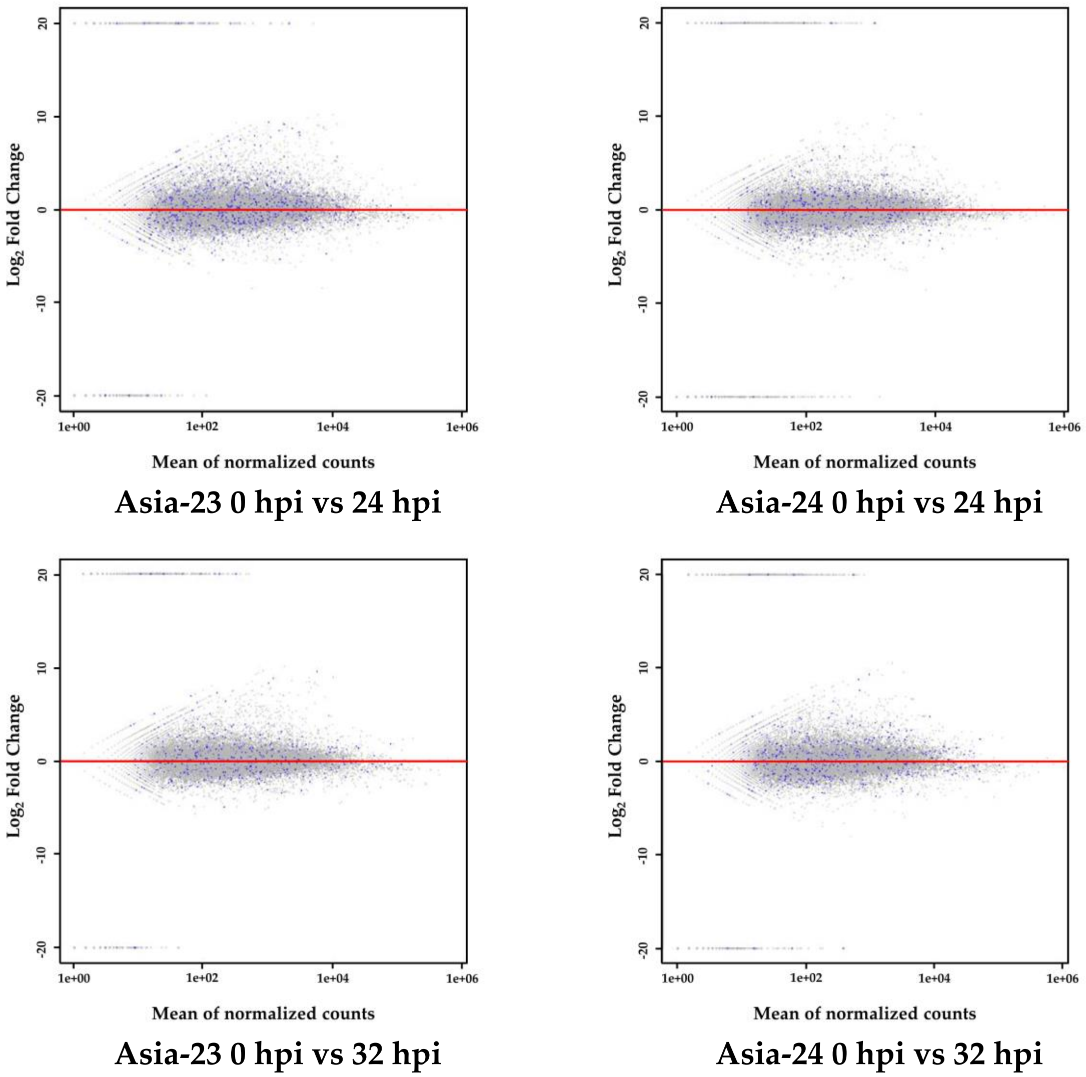

Figure S2. Differentially expressed gene (DEG) results expressed as MA plot. In the MA plot, transcripts of significantly upregulated or downregulated genes in each comparative analysis ($p < 0.01$) are indicated with blue dots. hpi: hours post inoculation.

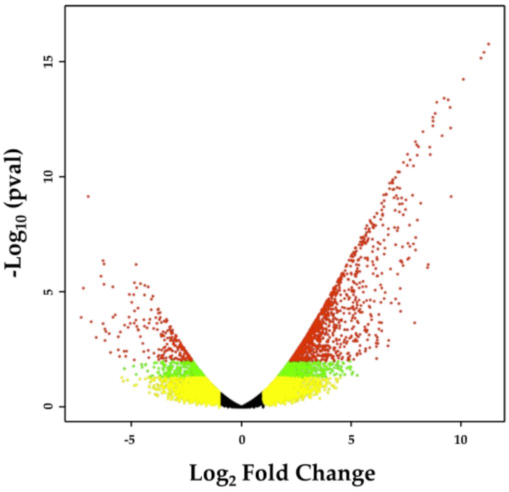

Asia-23 0 hpi vs 4 hpi

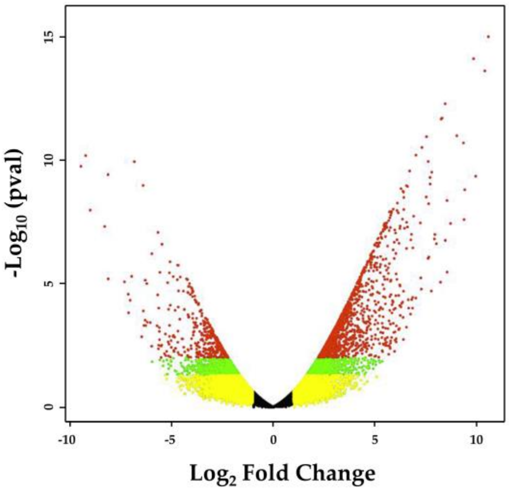

Asia-24 0 hpi vs 4 hpi

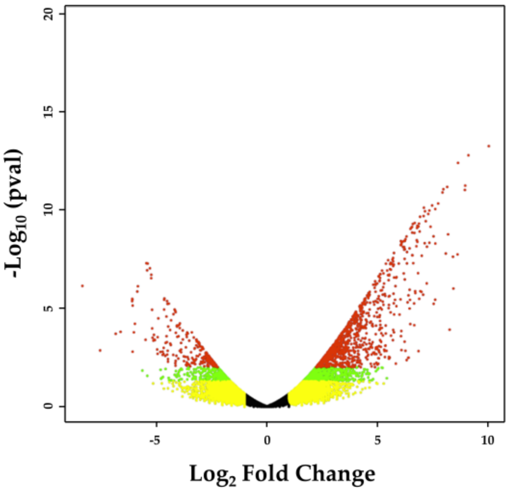

Asia-23 0 hpi vs 8 hpi

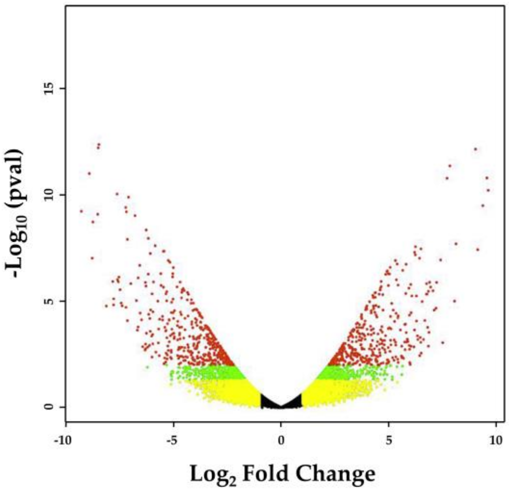

Asia-24 0 hpi vs 8 hpi

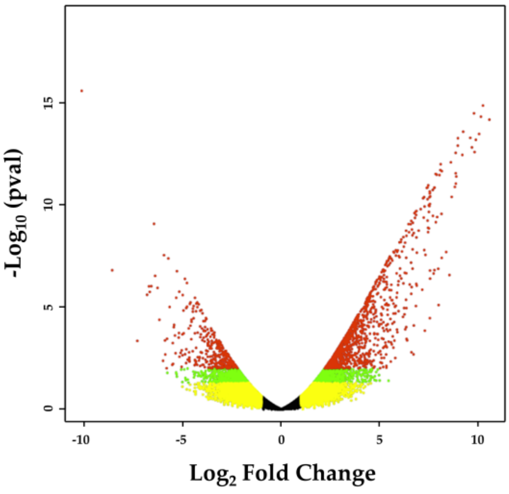

Asia-23 0 hpi vs 16 hpi

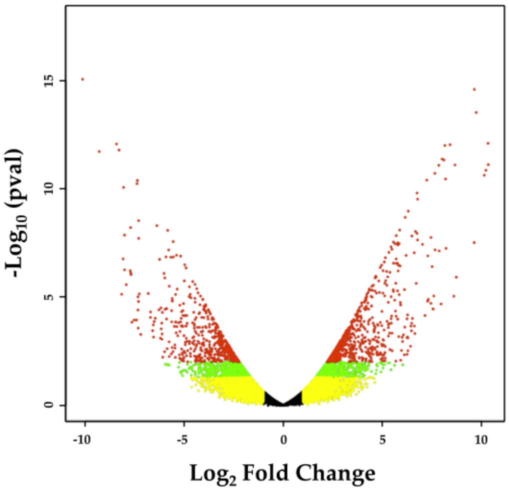

Asia-24 0 hpi vs 16 hpi

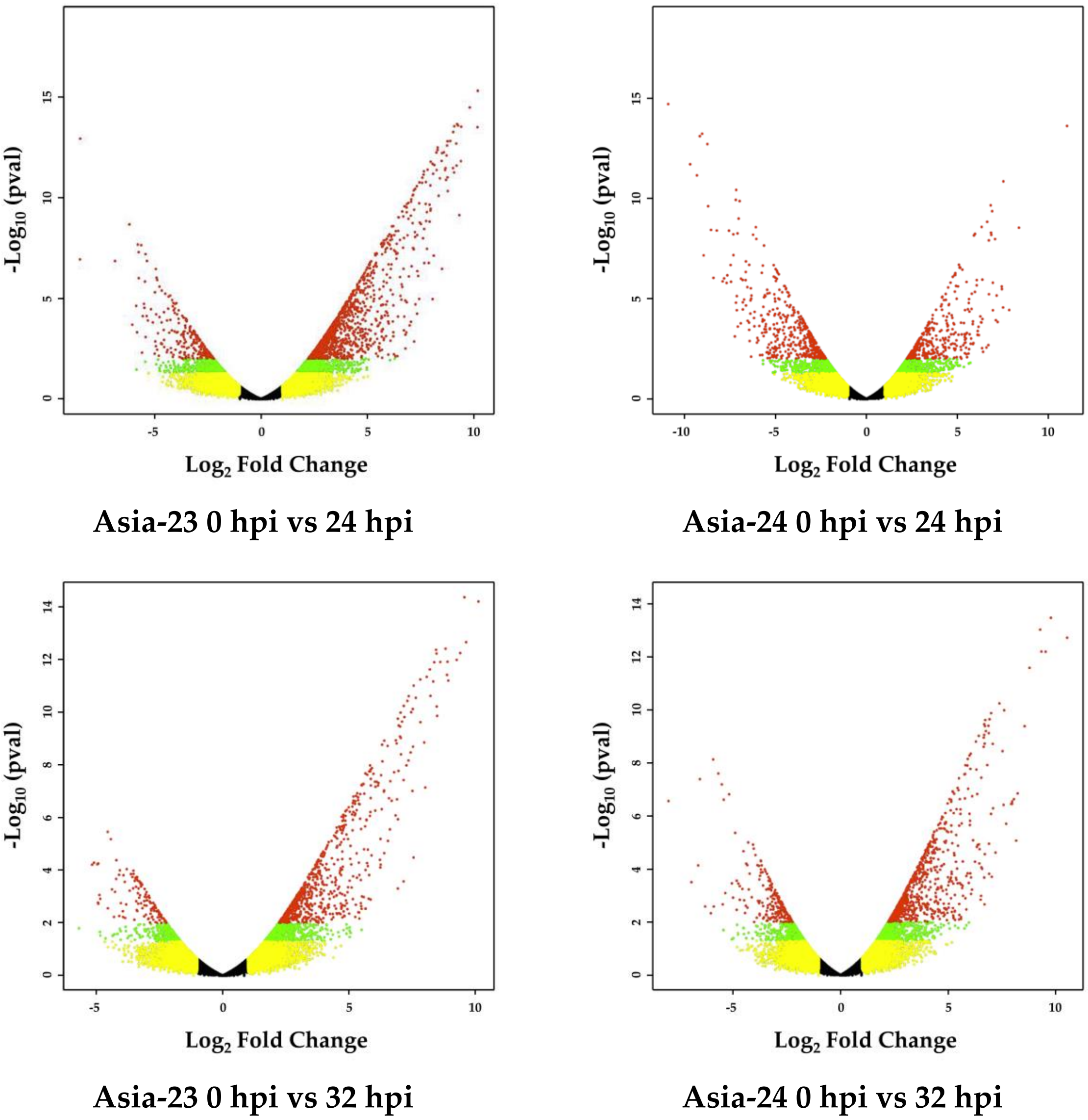

Figure S3. Differentially expressed gene (DEG) results expressed as Volcano plot. The Volcano plots were displayed in different colors according to each analysis option in the DEG analysis: FDR < 0.01, 2 < |log2 fold cha2nge| (red), 0.01 < FDR < 0.05, 2 < |log2 fold change| (green), 0.05 < FDR, 2 < |log2 fold change| (yellow), and FDR < 0.05, |log2 fold change| < 2 (black). hpi: hours post inoculation.

(A)

GO Cellular Component

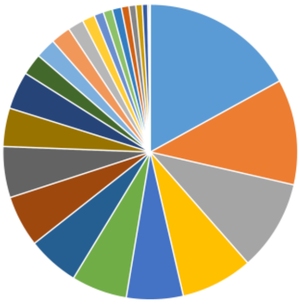

Functional category	Gene count
nucleus	1772
chloroplast	1204
cytoplasm	1034
other intracellular components	822
plasma membrane	648
mitochondrion	636
cytosol	591
plastid	585
other membranes	582
other cellular components	438
extracellular region	430
vacuole	246
Golgi apparatus	230
endoplasmic reticulum	226
thylakoid	181
unknown cellular components	143
nucleolus	108
nucleoplasm	105
cell wall	105
peroxisome	87
endosome	78
cytoskeleton	73
ribosome	62
nuclear envelope	19
lysosome	10

(B)

GO Molecular Function

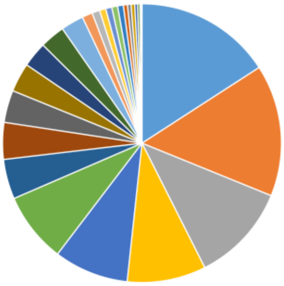

Functional category	Gene count
protein binding	1170
catalytic activity	1127
unknown molecular functions	842
transferase activity	674
other binding	638
hydrolase activity	601
RNA binding	341
DNA binding	315
transporter activity	276
kinase activity	248
nucleic acid binding	216
DNA-binding transcription factor activity	214
nucleotide binding	195
enzyme regulator activity	89
structural molecule activity	66
nuclease activity	54
chromatin binding	52
other molecular functions	50
lipid binding	49
carbohydrate binding	35
transcription regulator activity	31
translation factor activity, RNA binding	31
signaling receptor activity	24
motor activity	21
signaling receptor binding	10
translation regulator activity	5
oxygen binding	1

(C)

GO Biological Process

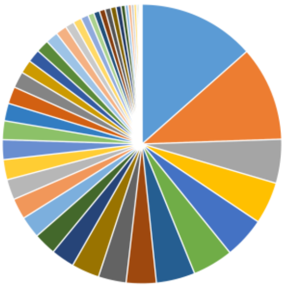

Functional category	Gene count
other cellular processes	2913
other metabolic processes	2368
anatomical structure development	1099
response to stress	1048
biosynthetic process	1032
response to chemical	1012
multicellular organism development	972
nucleobase-containing compound metabolic process	750
cellular component organization	696
reproduction	696
post-embryonic development	582
response to abiotic stimulus	563
cellular protein modification process	547
response to external stimulus	520
catabolic process	510
response to light stimulus	503
response to endogenous stimulus	496
transport	469
response to biotic stimulus	439
signal transduction	426
unknown biological processes	407
other biological processes	336
protein metabolic process	324

Functional category	Gene count
lipid metabolic process	320
growth	310
cell differentiation	276
carbohydrate metabolic process	220
embryo development	209
cell cycle	191
cell communication	149
DNA metabolic process	148
flower development	144
secondary metabolic process	140
cell growth	137
translation	125
generation of precursor metabolites and energy	101
pollination	82
photosynthesis	72
regulation of molecular function	66
cellular homeostasis	62
cell death	39
circadian rhythm	38
tropism	27
regulation of gene expression, epigenetic	19
cell-cell signaling	10
abscission	6

Figure S4. GO annotation of transcripts clustered in Cluster 4. (A) GO Cellular Component, (B) GO Molecular Function, (C) GO Biological Process.

(A)

Alpha-linolenic acid metabolism pathway

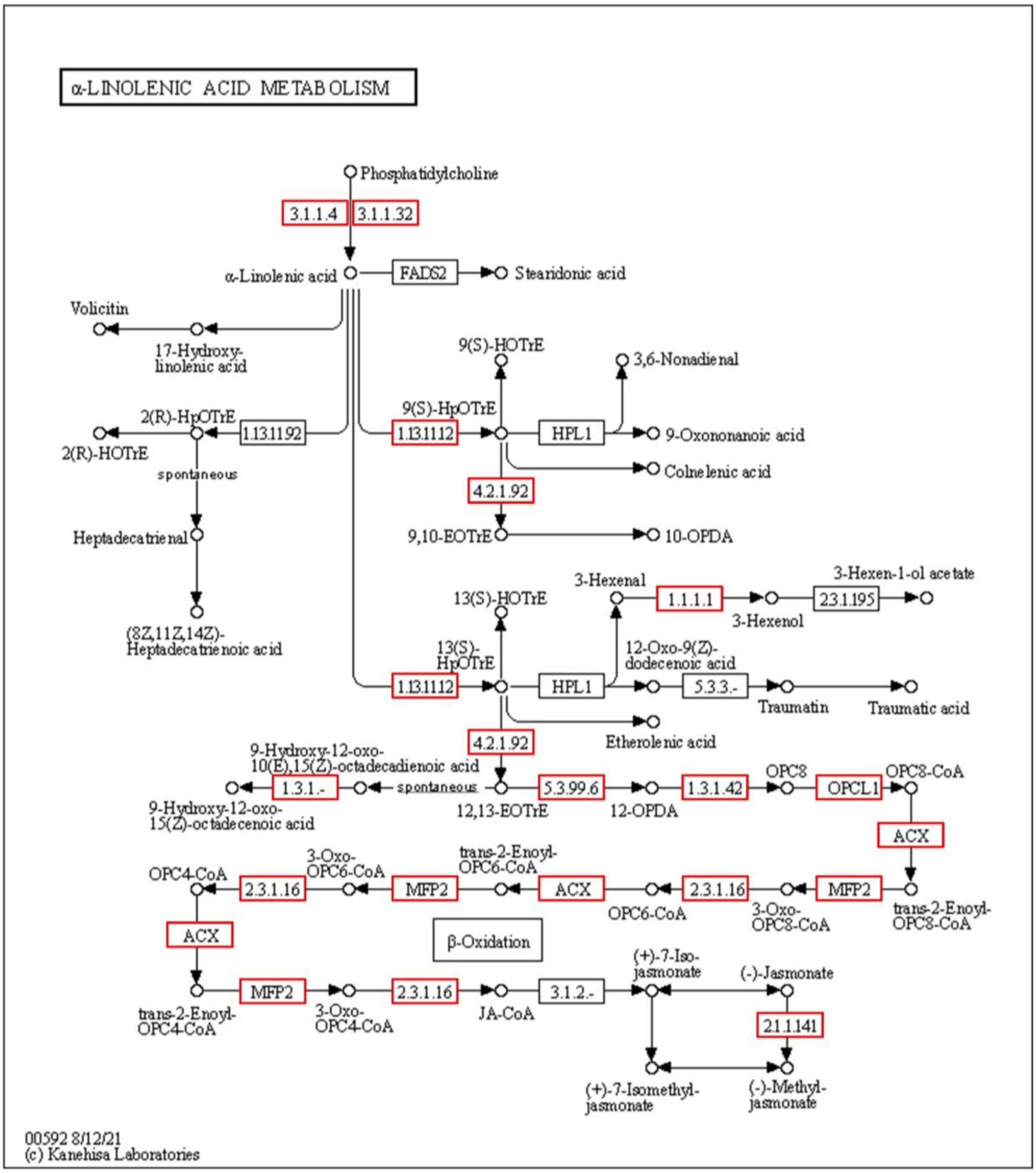

(B)

MAPK signaling pathway

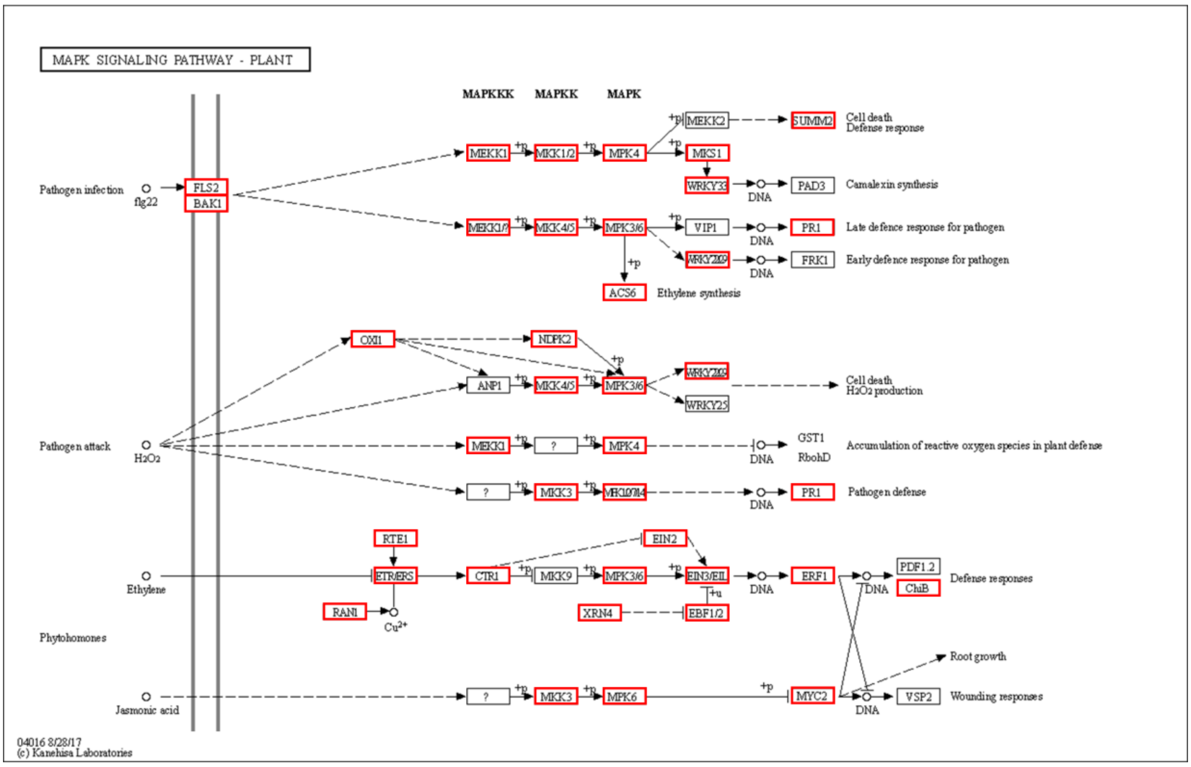

(C)

Plant hormone signal transduction – Carotenoid biosynthesis pathway

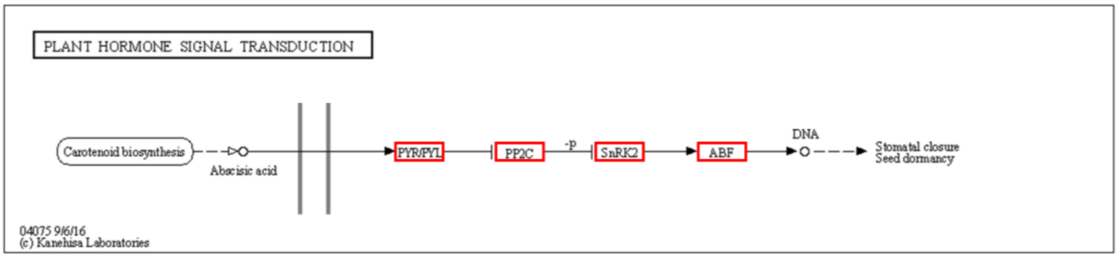

Plant hormone signal transduction – Cysteine and methionine metabolism

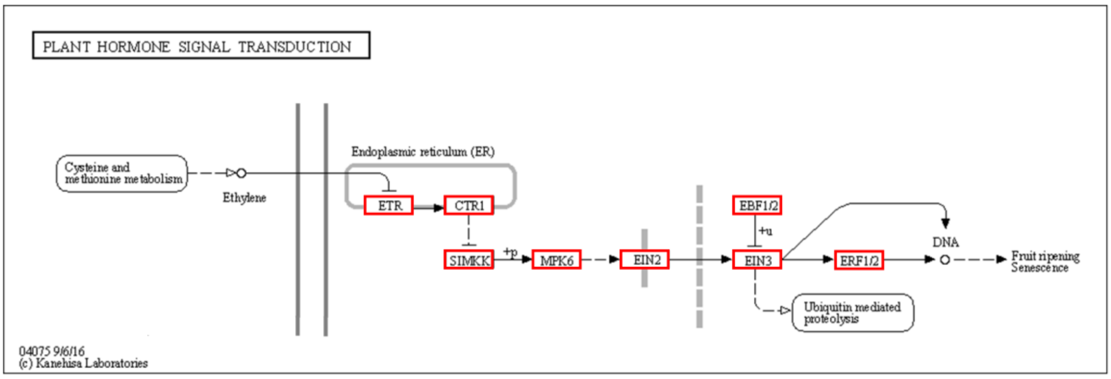

Plant hormone signal transduction – Brassionosteroid biosynthesis

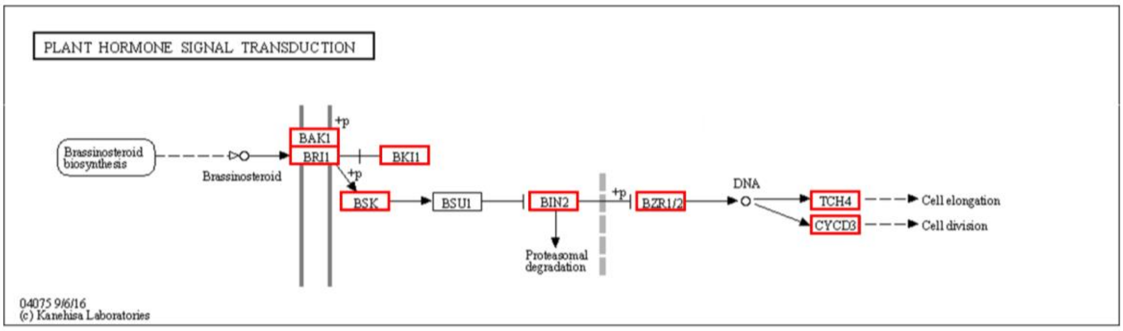

Plant hormone signal transduction – Phenylalanine metabolism

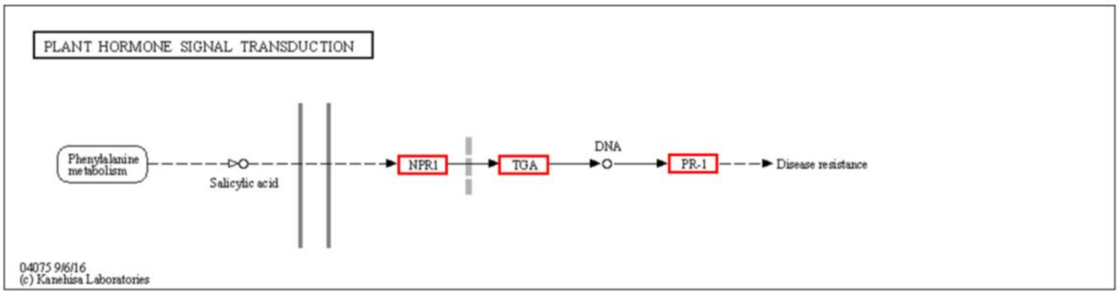

(D)

Plant – Pathogen interaction

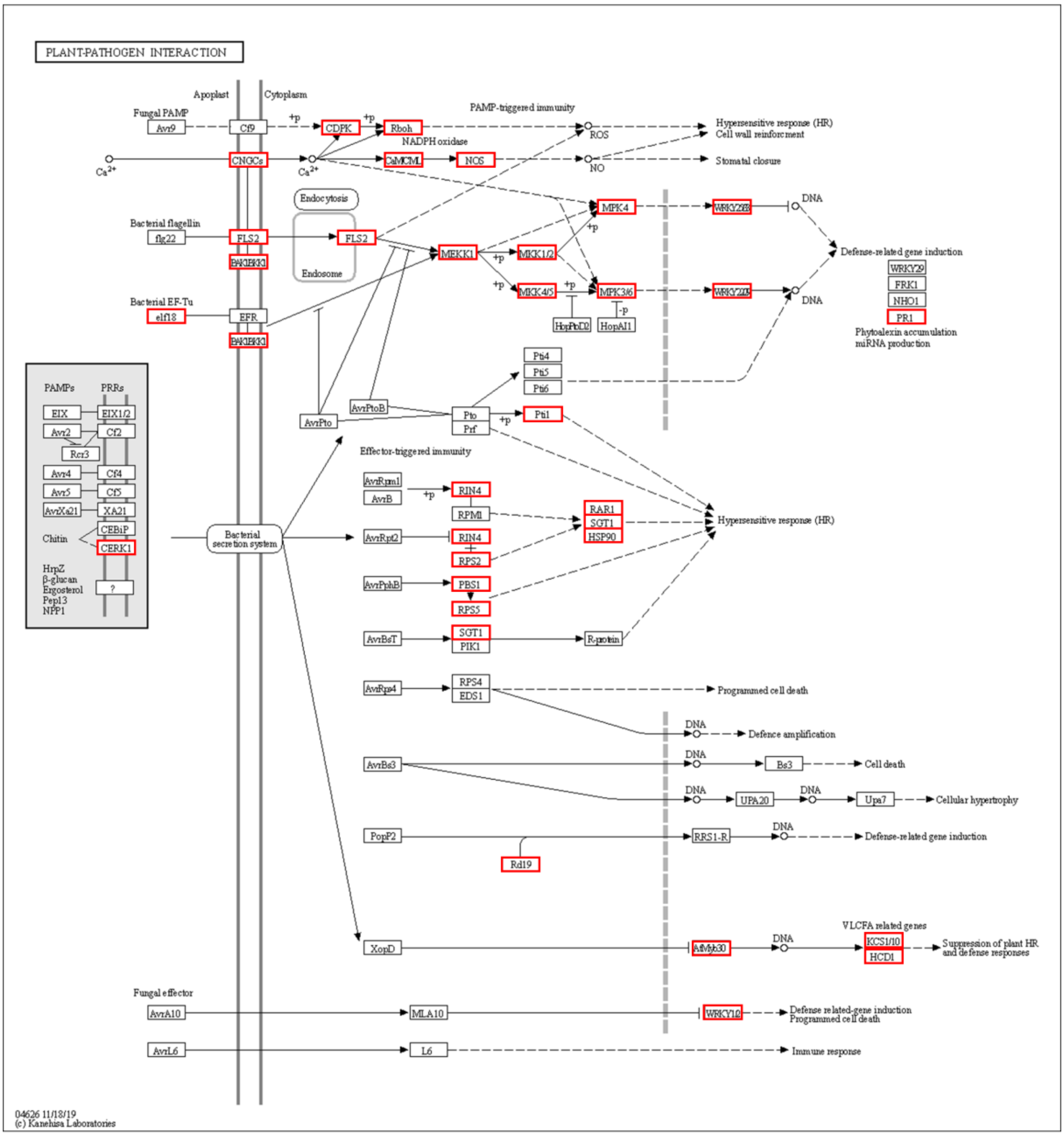

Figure S5. Results of KEGG pathway anaysis considered to be related to gray mold resistance. (A) Alpha-linolenic acid metabolism, (B) MAPK signaling pathway, (C) Plant hormone signal transduction-Carotenoid biosynthesis pathway; Plant hormone signal transduction-Cysteine and methionine metabolism; Plant hormone signal transduction-Brassionosteroid biosynthesis; Plant hormone signal transduction-Phenylalanine metabolism, (D) Plant-pathogen interaction. The genes matched with onion transcripts were displayed red box.

Table S1. Primer sets for confirmation of selected gene sequences

Gene	Primer name	Orientation	Nucleotide sequences (5′ to 3′)
JAR1	1st exon (Include start codon)	Forward	TGT TTT CCG CAG ACC TTT TGA A
		Reverse	TGG CTT CCC TTT TGT AGT ACC A
	2nd exon	Forward	AGC TGC CTT CAG TCA TCA CAA
		Reverse	CAC TAC ACT TGT GAC ATG TTC CG
	3rd exon (Include stop codon)	Forward	TCT ACG AGA AGG GGT AGC TGT
		Reverse	AGA ATA CGA ACA AAC CTG CAC C
COI1	1st exon (Include start codon)	Forward	TGC CGC TAG TAT AAT GTT GAG CT
		Reverse	ACT TGT ATC AGC TGC CCA GT
	2nd exon	Forward	GGA GGA GGC GGA AGT GTT TT
		Reverse	GCT CTG CTC CTC CCC TAA CA
	3rd exon (Include stop codon)	Forward	AGA GAT GTT TGC GTG CGA GT
		Reverse	ACA AAA CCA GCC AAG CAA ATT TTT A
MYC2	1st exon (Include start codon)	Forward	CTC GAC GCA CTA ACC GGA G
		Reverse	TGC TAA AAC AAC GCT CGA TCA
	2nd exon (Include stop codon)	Forward	CCT TCT CCT TCT ACC TCA TTT CCT C
		Reverse	CAT ATC TAA AAG TCC GTC TTC TCC G

Table S2. Primer sets for high-resolution melting (HRM) analysis.

Primer name	Orientation	Nucleotide sequences (5′ to 3′)
JAR1	Forward	GCT GAG TAT TTG CAA AAT TTG GGT C
	Reverse	TGT CAA TGG AGT TCC GGT CAG
COI1	Forward	GGC GGC GAT TTC CTA CGT TA
	Reverse	CCT GCA TAG CCT CCC CAA TC
MYC2	Forward	CAA CCA GGA ATC CCT CCA GC
	Reverse	GAC TGC CAG AAG ATG GCG TA

Table S3. Statistics of sequence raw data.

Sample ID	Total read bases	Total reads	Q30 [a] (%)
Asia_23_0 hpi	5,159,368,052	51,082,852	93.96
Asia_23_4 hpi	5,397,686,642	53,442,442	93.99
Asia_23_8 hpi	5,410,303,966	53,567,366	94.85
Asia_23_16 hpi	5,459,818,004	54,057,604	93.19
Asia_23_24 hpi	5,427,496,792	53,737,592	94.04
Asia_23_32 hpi	5,164,047,382	51,129,182	93.58
Asia_24_0 hpi	5,343,300,768	52,903,968	95.06
Asia_24_4 hpi	5,301,659,276	52,491,676	93.99
Asia_24_8 hpi	5,356,953,342	53,039,142	93.92
Asia_24_16 hpi	5,322,845,642	52,701,442	94.17
Asia_24_24 hpi	5,252,278,962	52,002,762	93.94
Asia_24_32 hpi	5,122,853,118	50,721,318	94.36

[a] Ratio of bases that have phred quality score greater than or equal to 30.

Table S4. Pre-processing results of onion RNA sequence raw data.

Sample ID	Raw Data		Trimmed Data	
	Total Reads	Total read bases (bp)	Total Reads	Total read bases (bp)
**Asia-23_0_hpi**	53,442,442	5,397,686,642	48,159,176	4,846,678,416
**Asia-23_4_hpi**	53,567,366	5,410,303,966	49,467,140	4,979,339,740
**Asia-23_8_hpi**	54,057,604	5,459,818,004	47,964,876	4,824,642,022
**Asia-23_16_hpi**	53,737,592	5,427,496,792	49,021,478	4,932,839,546
**Asia-23_24_hpi**	51,129,182	5,164,047,382	45,008,816	4,529,201,024
**Asia-23_32_hpi**	51,947,444	5,246,691,844	46,223,388	4,651,745,224
**Asia-24_0_hpi**	52,903,968	5,343,300,768	48,938,402	4,927,956,063
**Asia-24_4_hpi**	52,491,676	5,301,659,276	47,029,632	4,732,586,446
**Asia-24_8_hpi**	53,039,142	5,356,953,342	47,633,440	4,793,750,309
**Asia-24_16_hpi**	52,701,442	5,322,845,642	47,396,334	4,770,861,379
**Asia-24_24_hpi**	52,002,762	5,252,278,962	46,667,746	4,696,488,290
**Asia-24_32_hpi**	50,721,318	5,122,853,118	45,931,580	4,623,373,129

Table S5. Results of mapping and alignment to reference data of pre-processed RNA sequence data.

Sample	Progressed reads	Mapped reads	Mapping rate[a] (%)
**Asia-23_0_hpi**	48,159,176	40,345,980	83.80%
**Asia-23_4_hpi**	49,467,140	36,736,709	74.30%
**Asia-23_8_hpi**	47,964,876	37,878,527	79.00%
**Asia-23_16_hpi**	49,021,478	40,383,170	82.40%
**Asia-23_24_hpi**	45,008,816	38,654,847	85.90%
**Asia-23_32_hpi**	46,223,388	39,394,150	85.20%
**Asia-24_0_hpi**	48,938,402	40,505,969	82.80%
**Asia-24_4_hpi**	47,029,632	37,595,701	79.90%
**Asia-24_8_hpi**	47,633,440	36,243,746	76.10%
**Asia-24_16_hpi**	47,396,334	37,886,277	79.90%
**Asia-24_24_hpi**	46,667,746	38,889,514	83.30%
**Asia-24_32_hpi**	45,931,580	38,286,473	83.40%

[a] Mapping rate: (Mapped reads / Progressed reads) x 100

Table S6. DEG comparison at each hour post inoculation (hpi) within the resistant and susceptible sample groups.

DEG group	Upregulated genes	Downregulated genes	Total
**Asia_23_0 hpi vs Asia_23_4 hpi**	770	90	860
**Asia_23_0 hpi vs Asia_23_8 hpi**	535	73	608
**Asia_23_0 hpi vs Asia_23_16 hpi**	677	136	813
**Asia_23_0 hpi vs Asia_23_24 hpi**	673	101	774
**Asia_23_0 hpi vs Asia_23_32 hpi**	317	37	354
**Asia_24_0 hpi vs Asia_24_4 hpi**	670	166	836
**Asia_24_0 hpi vs Asia_24_8 hpi**	476	131	607
**Asia_24_0 hpi vs Asia_24_16 hpi**	761	300	1061
**Asia_24_0 hpi vs Asia_24_24 hpi**	340	112	452
**Asia_24_0 hpi vs Asia_24_32 hpi**	406	58	464
